# *Arabidopsis* CALMODULIN-LIKE 38 Regulates Hypoxia-Induced Autophagy of SUPPRESSOR OF GENE SILENCING 3 Bodies

**DOI:** 10.3389/fpls.2021.722940

**Published:** 2021-09-08

**Authors:** Sterling Field, William Craig Conner, Daniel M. Roberts

**Affiliations:** Department of Biochemistry & Cellular and Molecular Biology, The University of Tennessee, Knoxville, TN, United States

**Keywords:** mRNP granules, stress granules, calmodulin, submergence stress, plant hypoxia, flooding, siRNA bodies, autophagy

## Abstract

During the energy crisis associated with submergence stress, plants restrict mRNA translation and rapidly accumulate stress granules that act as storage hubs for arrested mRNA complexes. One of the proteins associated with hypoxia-induced stress granules in *Arabidopsis thaliana* is the calcium-sensor protein CALMODULIN-LIKE 38 (CML38). Here, we show that SUPPRESSOR OF GENE SILENCING 3 (SGS3) is a CML38-binding protein, and that SGS3 and CML38 co-localize within hypoxia-induced RNA stress granule-like structures. Hypoxia-induced SGS3 granules are subject to turnover by autophagy, and this requires both CML38 as well as the AAA^+^-ATPase CELL DIVISION CYCLE 48A (CDC48A). CML38 also interacts directly with CDC48A, and CML38 recruits CDC48A to CML38 granules in planta. Together, this work demonstrates that SGS3 associates with stress granule-like structures during hypoxia stress that are subject to degradation by CML38 and CDC48-dependent autophagy. Further, the work identifies direct regulatory targets for the hypoxia calcium-sensor CML38, and suggest that CML38 association with stress granules and associated regulation of autophagy may be part of the RNA regulatory program during hypoxia stress.

## Introduction

In response to low oxygen stress associated with submergence and flooding, *Arabidopsis thaliana* reprioritizes gene expression to favor a small set of “core” hypoxia-induced genes that encode various proteins that coordinate an adaptive response (Mustroph et al., 2009; Lee and Bailey-Serres, 2021). CALMODULIN-LIKE 38 (CML38) is a calcium-sensor protein that is one of 49 core hypoxia response proteins in *A. thaliana*. CML38 is acutely induced early in hypoxia and localizes in cytosolic foci that accumulate during low oxygen stress (Lokdarshi et al., 2016). Characterization of CML38-associated proteins by immunoprecipitation and mass spectrometry revealed several stress granule (SG) markers as well as various proteins associated with RNA processing and metabolism (Lokdarshi et al., 2016). Further, transient transfection analyses in *Nicotiana benthamiana* revealed that CML38 co-localizes with a stress granule marker, RBP47B (Lokdarshi et al., 2016). The accumulation of stress granules during hypoxia stress is a hallmark of the response in *A. thaliana* (Sorenson and Bailey-Serres, 2014), and is associated with a general arrest of energetically expensive translation, a decrease in translating polysomes (Branco-Price et al., 2008), and the accumulation of arrested preinitiation mRNA complexes (Branco-Price et al., 2005, 2008). It was postulated that CML38 is a hypoxia core-response protein that is a component of hypoxia-induced mRNA stress granules (Lokdarshi et al., 2016).

While CML38 is necessary for an optimal response of *A. thaliana* to low oxygen stress (Lokdarshi et al., 2016), its functional role as a component of hypoxia-induced RNA granules is less clear. CML38 is structurally and phylogenetically similar to a *Nicotiana* protein known as “Regulator of gene silencing calmodulin” (rgsCaM). rgsCaM was originally identified in *Nicotiana tabacum* as a binding target of the potyviral RNA silencing protein “helper component proteinase” (HC-Pro; Anandalakshmi et al., 2000). rgsCaM overexpression suppresses secondary siRNA gene silencing (Anandalakshmi et al., 2000), and it was proposed that rgsCaM is an endogenous suppressor of Post Transcriptional Gene Silencing (PTGS), and that viral HC-Pro may take advantage of this physiological function to evade the plant host response (Anandalakshmi et al., 2000).

A potential mechanism for rgsCaM suppression of host gene siRNA silencing came from the observation that the SUPPRESSOR OF GENE SILENCING 3 (SGS3) RNA binding protein is a direct interaction target for rgsCaM (Li et al., 2017). *SGS3* encodes an RNA-binding homodimeric protein that, together with the RNA-dependent RNA polymerase 6 (RDR6), is necessary for the synthesis of dsRNA templates for secondary siRNA production in viral, transposon, and transgene PTGS pathways (Mourrain et al., 2000; Peragine et al., 2004; Yoshikawa et al., 2005; Kim et al., 2021). SGS3 accumulates within cytosolic foci termed siRNA bodies that are proposed to be involved in secondary siRNA biosynthesis (Elmayan et al., 2009; Kumakura et al., 2009; Jouannet et al., 2012). Overexpression of rgsCaM lowers the numbers of siRNA bodies as well as the level of SGS3 protein (Li et al., 2017). This effect of rgsCaM overexpression was suppressed by autophagy inhibitors and RNA_i_ reduction of the expression of key autophagy genes (Li et al., 2017).

In the present work, we demonstrate that SGS3 is also a direct binding target for *A. thaliana* CML38, that CML38 and SGS3 co-localize to hypoxia-induced cytosolic granules, and that CML38 regulates SGS3 and granule autophagy during sustained hypoxia. Moreover, we demonstrate that CML38 interacts with the ubiquitin segregase AAA^+^-ATPase CDC48, and that this interaction appears to be necessary for SGS3 granule autophagy during hypoxia.

## Materials and Methods

### Plant Growth and Stress Treatments

All lines in this work were generated in the *A. thaliana* Colombia-0 (Col-0) background. The following mutant lines were generated and characterized as previously described: *cml38* (Lokdarshi et al., 2016), *sgs3-11* (Peragine et al., 2004), and *atg9-4* (Floyd et al., 2015). *Arabidopsis thaliana* seeds were surface sterilized and plated onto ½ strength Murashige and Skoog media, pH 5.6, without sucrose (½MS). Seeds were vernalized for 48h at 4°C, and then were germinated and grown under long day growth conditions [16h light (~100μmolm^−2^ s^−1^)/8h dark] at 22°C. Experimental treatments were performed with 10-day old seedlings at 8h into the light cycle. Hypoxia was induced by challenging seedlings with argon gas (AR UHP300, Airgas; <2% oxygen, measured using Traceable Oxygen probe, Fisher Scientific) under light conditions (70μmolm^−2^ s^−1^). Autophagy inhibitor treatments were performed by supplementing media with 10μM E64d (UBPBio), 5μM wortmannin (LC Laboratories), or 1μM concanamycin A (Santa Cruz Biotechnologies) by the approach described by Merkulova et al. (2014). CDC48 inhibitor treatments were performed by supplementing media with 2μM CB5083 (ApexBio) as described by Marshall et al. (2019).

Selected hypoxia experiments were performed on the roots of hydroponic *A. thaliana* plants by the method described in Lokdarshi et al. (2016). *Arabidopsis thaliana* plants were grown in ½MS with aeration under short day conditions [8h light (~100μmolm^−2^ s^−1^)/16h dark] at 22°C as described in Gibeaut et al. (1997). Hypoxia treatments were performed on 40-day old plants by replacing the air supply with nitrogen gas, and monitoring the dissolved oxygen level as described in Lokdarshi et al. (2016).

*Nicotiana benthamiana* plants for transient transfection and co-localization analyses were grown on Lambert LM-AP soil under long day conditions at 23°C. For hypoxia treatments, *N. benthamiana* 1cm leaf disks were dissected, transferred to ½MS media, and challenged with argon gas as described above. Submergence stress of leaf disks was done as described in Weber et al. (2008); Sorenson and Bailey-Serres (2014); Lokdarshi et al. (2016) by immersion in ½MS on a microscope slide under a coverslip and visualization after 1–2h of submergence.

### Molecular Cloning

All PCR primers used are described in Supplementary Table S1, and a list of all constructs generated is found in Supplementary Table S2. Constructs of various fluorescent protein translational fusions were generated in the pEarleyGate plant expression vector system under the control of the CaMV35S promoter (Earley et al., 2006). The coding regions for the *ATG8e*, *SGS3*, and *CDC48A* were generated by RT-PCR. Total RNA was extracted from 6h hypoxic *A. thaliana* Col-0 seedlings by using E.Z.N.A. Plant RNA Kit (OMEGA), and cDNA was synthesized using random hexamers with the High Capacity cDNA Reverse Transcription Kit, following the manufacturer’s protocol (Applied Biosystems). *eYFP*-*ATG8e*, *SGS3-CFP*, and *CDC48A-CFP* were generated by first amplifying the respective coding regions without their stop codons with gene-specific primers that incorporated the *attB1* and *attB2* or *attB4* Gateway (Invitrogen) recombination sites. The PCR products were recombined with the pDONR 207 donor plasmid in a BP Clonase II reaction according to manufacturer’s protocols (Invitrogen). pDONR 207 *SGS3* and pDONR 207 *CDC48A* were recombined into pEarleyGate 102 by using LR Clonase II (Invitrogen). The pEarleyGate 102 *RBP47B-CFP* and pEarleyGate 101 *CML38-YFP* reporter constructs were generated as described previously (Lokdarshi et al., 2016).

A translational fusion construct of *RBP47B* with the *mScarlet* fluorescent reporter protein (Bindels et al., 2017) under the control of the *35SCaMV* promoter was prepared in pBin19 (Frisch et al., 1995). *Xba*I and *Xma*I sites were introduced into the *RBP47B* open reading frame at the 5' and 3' ends to facilitate cloning into pAN990 (a gift from Andreas Nebenführ, University of Tennessee – Knoxville) to generate the *mScarlet* translational fusion under the control of the *35SCaMV* promoter. A fragment containing *35S::RBP47B-mScarlet* was excised by digestion with *Sac*I and *Hind*III, and subcloned into pBin19.

For suppression of silencing assays in *N. benthamiana* 16C, the open reading frames of *CML38*, *rgsCaM*, and *HC-Pro* were cloned into pEarleyGate 100 under the CaMV35S promoter by a similar approach. Gene specific primers for *CML38*, *rgsCaM*, and *HC-Pro* incorporated *attB1* and *attB2* recombination sites. The *CML38* open reading frame was generated by RT-PCR using total RNA from 6h hypoxia *A. thaliana* Col-0 seedlings. The *rgsCaM* open reading frame was generated using total RNA from whole *N. tabacum* seedlings by RT-PCR. *HC-Pro* was generated by amplifying *Turnip Mosaic Virus Helper Component Proteinase* (*HC-Pro*) from pENTR *TuMV HC-Pro* (Endres et al., 2010).

For bacterial adenylate cyclase two hybrid (BACTH) analysis, the open reading frames of *CML38*, *CDC48A*, *eIF4A*, *GRP7*, *GRP8*, *DUF581-5*, and *SGS3* were generated by PCR amplification using the cDNA template generated from 6h hypoxia treated *A. thaliana* seedlings described above. PCR primers were engineered with an *Xba*1 site on the forward primer and a *Kpn*1 site on the reverse primer to allow insertion into the *Kpn*1 and *Xba*1 sites of pUT18c (CML38 bait) or pKNT25 plasmids (prey constructs; Karimova et al., 2000). Constructs were transformed into Top10 chemically competent *Escherichia coli* (Invitrogen), and positive transformants were selected on LB media supplemented with 50μg/ml carbenicillin (for pUT18C) or 25μg/ml kanamycin (for pKNT25).

All constructs were verified by Sanger DNA sequencing performed on an Applied Biosystems 3,730 Genetic Analyzer at the University of Tennessee Genomics Core Facility, Knoxville TN.

### Transfection Techniques

All reporter and silencing suppression constructs were transformed into *Agrobacterium tumefaciens* strain GV3101 (Koncz and Schell, 1986) by electroporation (Jones, 1995). The *35S:SGS3-CFP* reporter transgenic lines were generated in both *A. thaliana* Col-0 and the *cml38* background (Lokdarshi et al., 2016). *Arabidopsis thaliana* transformation was performed by the floral dip method (Clough and Bent, 1998). Seeds were selected on ½MS media with 25μg/ml glufosinate. T3 transgenic lines were used for all experiments.

Transient transfection was performed on leaves of 4–6-week old *N. benthamiana* plants using the general method of Wang et al. (2013). *A. tumefaciens* was diluted with infiltration buffer (10mM MES NaOH pH 5.7, 10mM MgCl_2_, 200μM acetosyringone) to an OD_600_ of 0.4 for single constructs, or to an OD_600_ of 0.8 (mixed 1:1) for co-expression constructs. Infiltration was performed with 0.5ml of these solutions by the method described in Lokdarshi et al. (2016). Transfected plants were grown at 20°C overnight, and were then used for hypoxia experiments starting the following day, imaging was performed ~48h after infiltration.

*N. benthamiana* 16C lines (Ruiz et al., 1998) were used for analysis of RNA silencing suppression. Leaves of 4-week old plants were infiltrated with 0.5ml (OD_600_ 0.4) *A. tumefaciens* GV3101 containing the silencing constructs of interest. After infiltration, plants were incubated at 21°C overnight, and then were moved back into long day growth conditions. Suppression of silencing was assessed by analysis of GFP signal under a UV light (UVGL-58 UV Lamp, UVP) 5days after infiltration.

### Microscopy and Imaging Methods

Imaging experiments were conducted with a Leica SP8 Confocal Microscope system (Wetzlar, Germany) at the Advanced Microscopy and Imaging Center, at the University of Tennessee, Knoxville. Epidermal cells from the *A. thaliana* root transition zone were imaged (~300μm x~150μm root section per frame) with a z-axis step size of 2μm for each optical section (z-series depth of 15–40μm). The pinhole setting was between 0.8 and 2 AU for all images. All images were collected using 4x line averaging with bidirectional laser scanning at a speed of 600Hz. The settings for excitation/emission were: 470nm/480–530nm (CFP), 514nm/540–575nm (YFP), 590nm/600–660nm (mSCARLET). For colocalization experiments, each channel was captured sequentially to prevent signal crosstalk. Standard deconvolution of images was performed with the Huygens Essentials software (Scientific Volume Imaging, Hilversum, The Netherlands) using the automatic deconvolution standard profile. For lightning deconvolution, the Leica Lightning Deconvolution system was used to optimize image capture settings for Z-axis step size, resolution and scan speed, and to maximize resolution. The adaptive deconvolution profile setting in the Leica Lightning Deconvolution package was used.

For particle quantitation in *A. thaliana*, images of single optical sections of root epidermal cells were analyzed in ImageJ using the built-in Particle Counter program (Schneider et al., 2012). Data were collected from between 40 and 60 cells per treatment replicate and represent a minimum of three independent biological experiments. Particles smaller than 0.1μm^2^ and larger than 10μm^2^ were excluded. Statistical analysis was done in GraphPad Prism 8.01 using built-in programs (one-way ANOVA, two-way ANOVA, or multiple *t*-test analysis, described in each figure legend). Outliers were assessed by Tukey’s interquartile range analysis. Violin plots of data were generated by using the “show all points” option in Prism with each plot containing bars indicating the median, and the first and third quartile values.

For *N. benthamiana* experiments, 5–10 optical sections (1μm per section) were combined into a single image, and a 40μm by 40μm section was analyzed. Co-localization was measured by calculating Manders coefficients (Manders et al., 1993) by using the Just Another Colocalization Plugin (JACoP) plugin in ImageJ (Bolte and Cordelieres, 2006; Schneider et al., 2012).

### Immunochemical Methods

For SGS3 Western blot analysis, hydroponically grown *A. thaliana* plants were challenged with hypoxia, as described above, and root tissue was collected and frozen in liquid nitrogen. Tissue samples were ground to a fine powder with a mortar and pestle, and were extracted by resuspension (0.3g tissue/200μl) in 50mM Tris–HCl pH 7.5, 0.15M NaCl, 10% (v/v) glycerol, 0.01% (v/v) NP-40, 1mM dithiothreitol, protease inhibitor cocktail (Thermo Scientific, EDTA free, one tablet per 10ml of resuspension buffer). Samples were incubated on ice for 10min, and were then centrifuged at 16,000 × *g* for 15min at 4°C. The supernatant fraction (soluble extract) was collected and the protein concentration was measured by Bradford assay (Bio-Rad). Soluble extract protein (40μg total protein) was separated by SDS-PAGE on 12.5% (w/v) polyacrylamide gels, and electroblotted onto polyvinylidene difluoride membranes. Membranes were blocked with 5% (w/v) non-fat dry milk powder in 1x phosphate buffered saline with Tween 20 (PBST pH 7.4; 137mM NaCl, 27mM KCl, 10mM Na_2_PO_4_, 1.8mM KH_2_PO_4_, 0.05% (v/v) Tween 20) for 3h at 4°C. The membrane was then incubated with rabbit anti-SGS3 polyclonal antibodies (1μg/ml; AgriSera) for 16h at 4°C. The membrane was washed five times in 1x PBST (50ml/wash), and was then incubated with horseradish peroxidase (HRP)-coupled goat anti-rabbit IgG (0.05μg/ml; AgriSera) for 1h at 21°C. The membrane was washed five times in 1x PBST (50ml/wash), and chemiluminescent detection was performed as described in Wallace and Roberts (2005). SGS3-CFP Western blots were done by a similar protocol, but with the substitution of rabbit anti-GFP polyclonal antibodies (AgriSera) as the primary antibody.

### Bacterial Adenylate Cyclase Two-Hybrid

CML38 interaction with potential target proteins was analyzed by using the BACTH System kit following the manufacturer’s protocol (Euromedex). *Escherichia coli* BTH101 cells were transformed with the specified prey (pKNT25) and bait (pUT18C) constructs followed by selection on LB media supplemented with 50μg/ml carbenicillin or 25μg/ml kanamycin. Positive colonies were identified by assaying for Lac^+^ and Mal^+^ phenotypes by plating on two indicator media: 1x M63 maltose agar media plates containing 40μg/ml 5-Bromo-4-chloro-3-indolyl-β-D-galactopyranoside (X-Gal); and MacConkey maltose agar media plates followed by culture at 30°C.

### Quantitative Real Time PCR

Quantitative Real Time PCR (Q-PCR) was done as previously described (Lokdarshi et al., 2016) with modifications. PCR was performed with PerfeCTa SYBR Green SuperMix (Quantabio) with 10ng of total cDNA per a 12.5μl reaction. Q-PCR was performed with a BioRad CFX96 Real-Time System and the following parameters: 1cycle of 5min at 95°C followed by 40cycles of: 15s at 95°C, 15s at 55°C, 30s at 72°C. Quantitation of gene expression was calculated using the comparative threshold cycle (Ct) method, as described in Choi and Roberts (2007). *UBQ10* was used as the reference gene for the calculation of ∆Ct in *A. thaliana*, *NbACTIN* was used as the reference gene for *N. benthamiana*. Primers used for Q-PCR are listed in Supplementary Table S1.

### Accession Numbers

Sequence data from this article can be found in the EMBL/GenBank data libraries under the following accession numbers: *CALMODULIN-LIKE 38* (*CML38*; AT1G76650), *SUPPRESSOR OF GENE SILENCING 3* (*SGS3*; AT5G23570), *CELL DIVISION CYCLE 48A* (*CDC48A*; AT3G09840), *GLYCINE RICH PROTEIN 7* (*GRP7*; AT2G21660), *GLYCINE RICH PROTEIN 8* (*GRP8*; AT4G39260), *EUKARYOTIC INITIATION FACTOR 4A* (*eIF4A*; AT1G54270), *DOMAIN OF UNKNOWN FUNCTION 581-5* (*DUF581-5*; AT1G74940), *RNA BINDING PROTEIN 47B* (*RBP47B*; AT3G19130), *RNA DEPENDENT RNA POLYMERASE 6* (*RDR6*; AT3G49500), *UBIQUITIN 10* (*UBQ10*; AT4G05320), *Nicotiana tabacum REGULATOR OF GENE SILENCING CALMODULIN* (*NtrgsCaM*; AF329729.1), *Turnip Mosaic Virus HELPER COMPONENT PROTEASE* (*HC-Pro*; EF028235), *Nicotiana benthamiana ACTIN* (*NbACTIN*; AY179605).

## Results

### Identification of Direct Interaction Protein Targets for CML38 by Bacterial BACTH

IP/MS experiments to identify the nature of CML38 foci in hypoxia-treated *A. thaliana* roots revealed an array of over 50 RNA metabolic proteins and RNP granule-associated proteins (Lokdarshi et al., 2016). These experiments, together with live cell imaging with granule markers, provided evidence for the association of CML38 with mRNA stress granules (Lokdarshi et al., 2016). These observations have been informative in revealing the collection of proteins that could be *in vivo* CML38 regulatory targets. However, since CML38 associates with mRNP complexes that contain multiple proteins, it is difficult to discern which are the direct binding/regulatory targets of CML38 and which are CML38 non-interacting proteins that are co-precipitated as part of the stress granule. Initial attempts to investigate CML38 interaction with selected protein targets by directed yeast two hybrid approaches were unsuccessful because of auto-activation by the CML38 bait construct. For this reason, a CML38 binding analysis utilizing the Bacterial Adenylate Cyclase-based Two-Hybrid (BACTH) system in *E. coli* was adopted (Karimova et al., 1998; Battesti and Bouveret, 2012).

**Figure 1:**
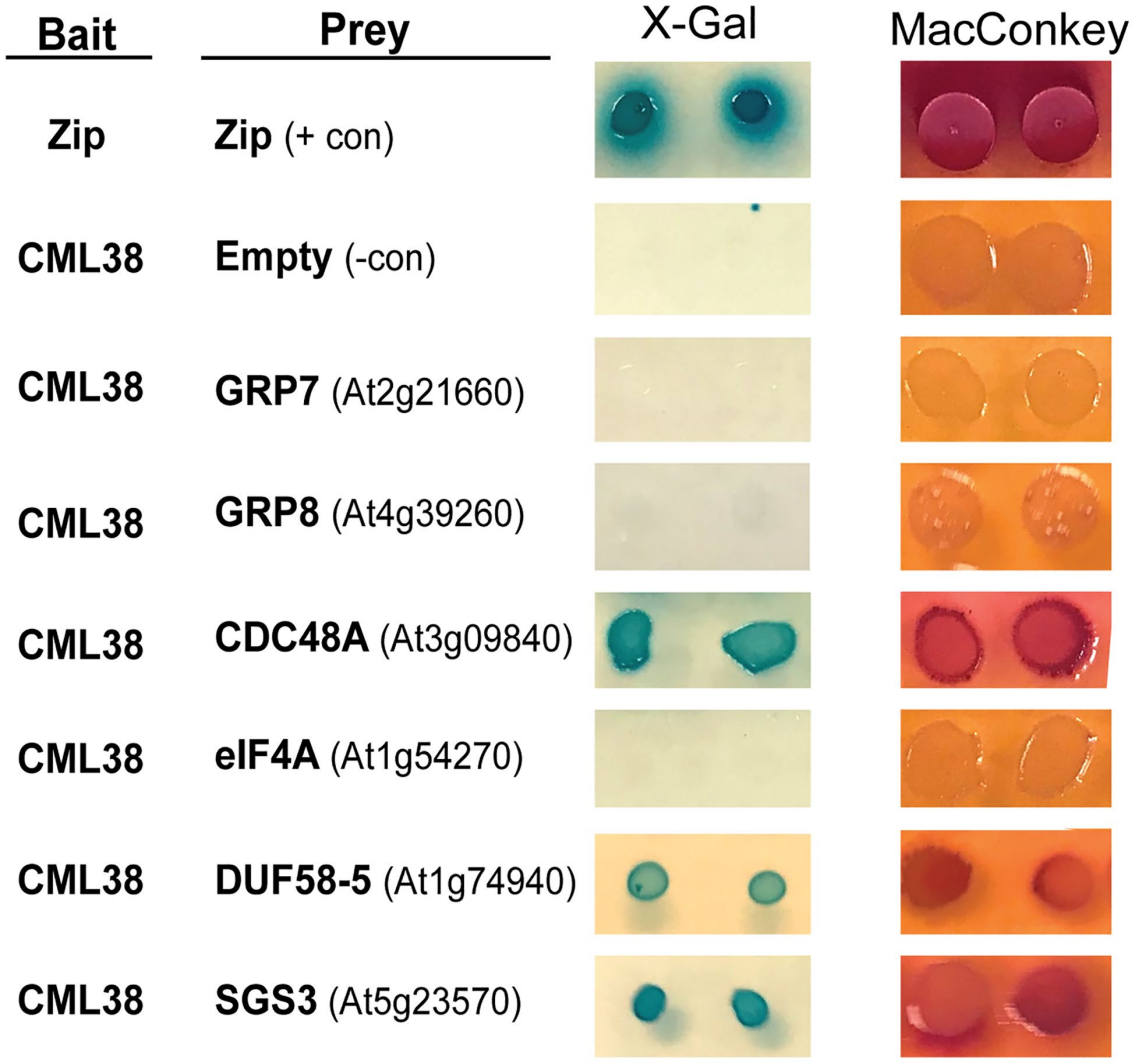
Analysis of CML38-interacting proteins by Bacterial Two Hybrid Assay. Bacterial Two Hybrid (BACTH) of CML38 with selected proteins of interest. *Escherichia coli* BTH101 cells were co-transformed with bait (*CML38*/pKNT25) and prey (protein of interest/pUT18c) constructs (see Materials and Methods). Gene names are listed and gene accession numbers are shown parenthetically. **Empty**, indicates a negative control consisting of the pUT18c construct without a protein fusion. **Zip**, indicates a positive control consisting of bait and prey constructs with two fragments of a leucine zipper motif of the yeast protein GCN4. Interaction analysis was performed by plating on M63 minimal media with the β-galactosidase substrate **X-Gal**, and a fermentation indicator **MacConkey** media supplemented with maltose, as described in the Materials and Methods.

The BACTH results with six representative proteins are shown in Figure 1. These include five proteins identified from CML38 IP pull down/MS experiments: the CDC48 AAA^+^-ATPase ubiquitin segregase; the RNA splicing proteins GRP8 and GRP7; the translation initiation factor RNA helicase eIF4A; and the SnRK1-interacting scaffold protein DUF581-5. We also analyzed a sixth protein, the SUPPRESSOR OF GENE SILENCING 3 (SGS3) RNA binding protein that is involved in siRNA biogenesis and is an interacting protein for *Nicotiana* rgsCaM (Li et al., 2017). Based on the analysis of two BACTH reporter assays (Figure 1), these proteins can be segregated into two classes: (1) direct CML38-interaction targets (e.g., DUF581-5, SGS3, and CDC48A); or (2) non-binding proteins (e.g., GRP7 and GRP8, and eIF4A) that likely were pulled down in IP experiments indirectly and do not bind to CML38.

### SGS3 Associates With CML38 in RNA Stress Granules in *Nicotiana benthamiana*

**Figure 2:**
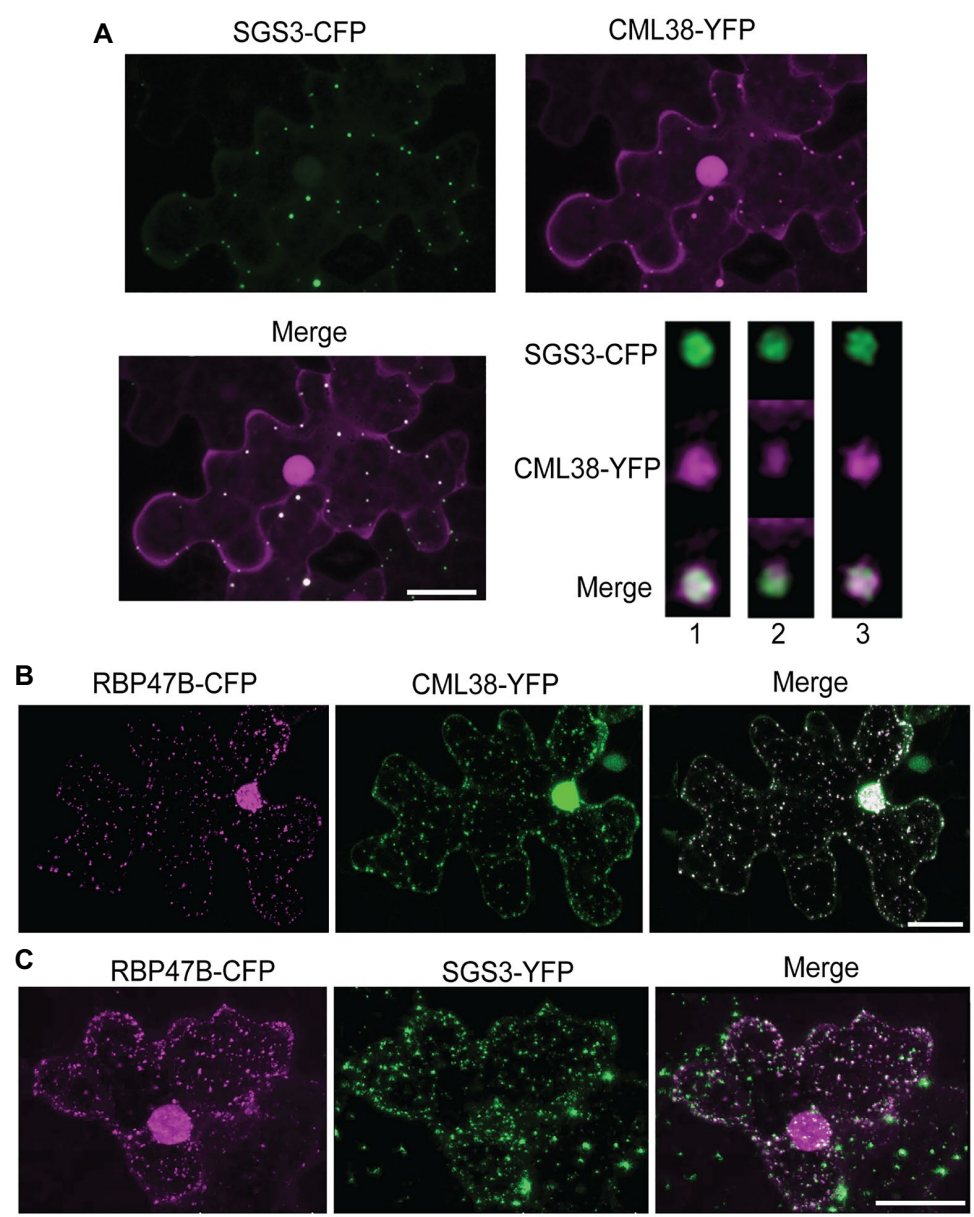
CML38 co-localizes with SGS3 and RBP47B granules. **(A)** Co-localization experiment with *Nicotiana benthamiana* leaves co-transfected with constructs that express SGS3-CFP and CML38-YFP. Leaf sections were subjected to submergence-induced hypoxia (3h) prior to imaging. The image is a 2D projection of 25 optical sections (0.8μm/section) and subjected to standard deconvolution as described in the Materials and Methods. The panel at the bottom right represents analysis of a single SGS3/CML38 granule showing three successive 0.23μm optical sections with Lightning deconvolution demonstrating co-localization of the two signals. **(B)** Co-localization experiment with *N. benthamiana* leaves co-transfected with constructs that express CML38-YFP and the stress granule marker RBP47B-CFP followed by submergence-induced hypoxia (1h) prior to imaging. The image is from 18 optical sections (2μm/section). **(C)** Co-localization experiment with *N. benthamiana* leaves co-transfected with SGS3-YFP and RBP47B-CFP. Leaf sections were subjected to submergence-induced hypoxia prior to confocal microscopy imaging. Scale bars are 25μm.

To explore the interaction of SGS3 and CML38 further, we performed co-localization experiments in transiently transfected *N. benthamiana* leaves (Figure 2). The submergence of *N. benthamiana* leaf disks in aqueous media induces a hypoxic state that leads to a time-dependent localization of CML38 to cytosolic stress granules (Lokdarshi et al., 2016). Previous work shows that SGS3 protein accumulates within other cytosolic foci termed siRNA bodies under non-stressed conditions, where it is proposed to be involved in trans-acting siRNA biosynthesis (Elmayan et al., 2009; Kumakura et al., 2009; Jouannet et al., 2012). SGS3 also strongly associates with stress granule marker proteins in response to heat stress (Jouannet et al., 2012). As shown in Figure 2A, CML38 and SGS3 show strong co-localization within cytosolic foci that form during submergence stress, including near complete signal superposition based on high-resolution analysis with deconvolution of z-stack images (Figure 2A). Each protein also shows strong co-localization with the RBP47B stress granule under these conditions (Figures 2B,C). Taken together with the BACTH data, the data suggest that CML38 and SGS3 interact in stress granules in response to submergence-induced hypoxia.

### Hypoxia Stress Induces Increases in SGS3 Body Formation Followed by CML38-Dependent Autophagy

**Figure 3:**
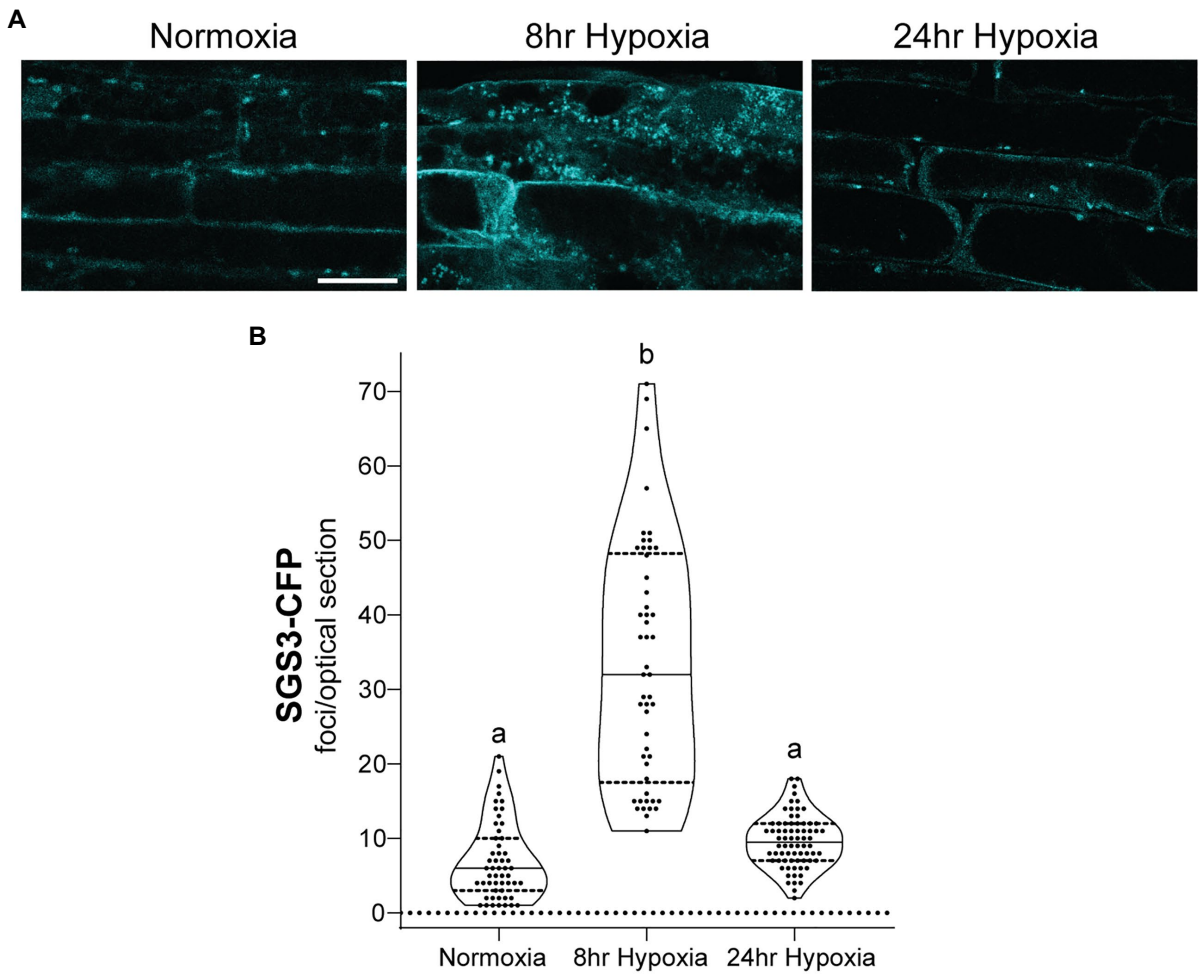
SGS3 body formation and turnover during hypoxia. **(A)** Confocal micrograph of representative *Arabidopsis thaliana* Col-0 epidermal root cells from *SGS3-CFP* plants during normoxia and hypoxia time course. All micrographs are the same scale. Scale bar is 25μm. **(B)** Violin plot of quantitation for the number of SGS3-CFP foci from A. Each data point represents quantitation of a 2μm optical section from a single cell. Different letters represent statistically significant differences (*p*<0.0001) assessed by one way ANOVA analysis.

To investigate the properties of SGS3 bodies in *A. thaliana*, and their potential regulation by CML38, stable transgenic lines expressing *SGS3-CFP* were generated in wildtype (Col-0) and *cml38* backgrounds. Confocal imaging of Col-0 *A. thaliana* root epidermal cells identified SGS3-CFP signal accumulating within some cytoplasmic bodies during normoxia (Figure 3), consistent with previous reports of SGS3-labeled siRNA bodies (Elmayan et al., 2009; Kumakura et al., 2009; Jouannet et al., 2012). During hypoxia, SGS3 bodies significantly increased in number, with a five-fold increase observed by 8h of hypoxia stress (Figure 3), similar to previous observations with stress granules (Sorenson and Bailey-Serres, 2014). However, upon extended hypoxia (24h), the number of SGS3 bodies declined (Figure 3) and were not statistically different from the level of SGS3 bodies under basal normoxic conditions.

**Figure 4:**
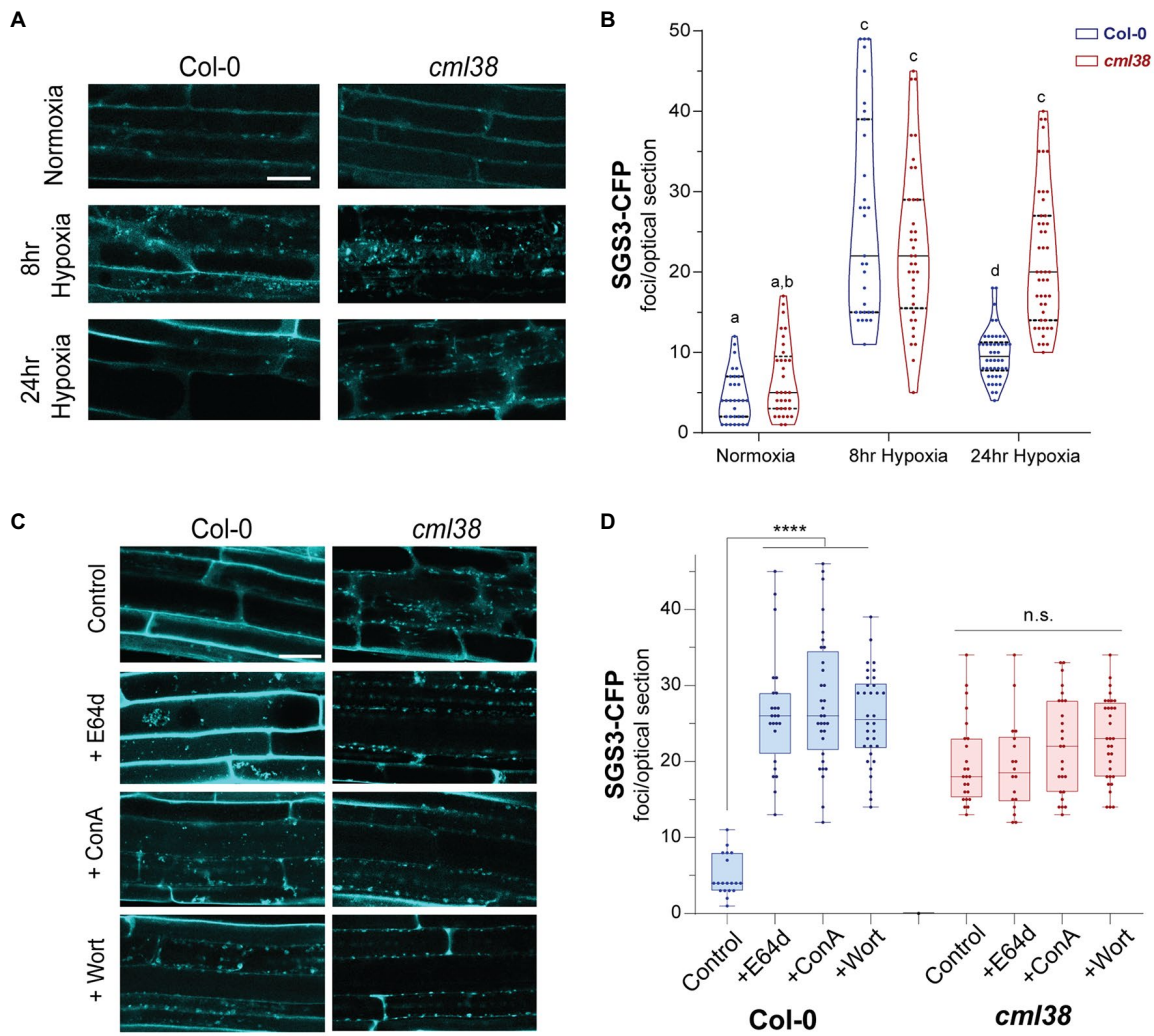
SGS3 requires CML38 and the autophagy pathway to be degraded during hypoxia. **(A)** Representative confocal micrographs of *A. thaliana* root epidermal cells in *SGS3-CFP* Col-0 and *cml38* transgenic plants challenged with hypoxia as described in the Materials and Methods. **(B)** Violin plot of quantitation of SGS3-CFP foci from **(A)**. Each data point represents a 2μm optical section from a single cell. Different letters represent statistically significant differences, assessed by one-way ANOVA analysis. **(C)** Representative confocal micrographs of *SGS3-CFP A. thaliana* Col-0 and *cml38* transgenic plants at 24h hypoxia or 24h hypoxia with the indicated autophagy inhibitors. **(D)** Quantitative analysis of SGS3-CFP foci from **(C)** as a box and whisker plot. Each data point represents a 2μm optical section from a single cell. Statistical significance was assessed by one-way ANOVA analysis. ****, *p*<0.0001; n.s., not significant. For (**A**,**C**), all micrographs are the same scale, and the scale bar is 25μm.

Next, the dynamics and time course of SGS3-CFP body accumulation was examined in the *cml38* background (Figure 4). Under normoxic conditions, Col-0 and *cml38* root epidermal cells did not show significant differences in the basal numbers of SGS3 bodies (Figure 4). Similarly the number of SGS3 bodies increased in a statistically indistinguishable manner during 8h hypoxia in both Col-0 and *cml38* cells (Figure 4). However, in contrast to Col-0, the number of SGS3 bodies in *cml38* remained elevated at 24h hypoxia, and did not significantly change from the 8h hypoxia timepoint (Figure 4B).

From this observation, the following questions emerged: (1) what is the nature of the SGS3 body disappearance under extended hypoxia; (2) why is this phenomenon lost in the *cml38* background? Since sustained submergence stress induces autophagy in *A. thaliana* (Chen et al., 2015), and stress conditions in yeast and mammals leads to a specialized selective autophagy of RNA granules known as “granulophagy” (Buchan et al., 2013; Wheeler et al., 2016), we investigated whether the decrease in SGS3 bodies during long term hypoxia might be the result of autophagy.

**Figure 5:**
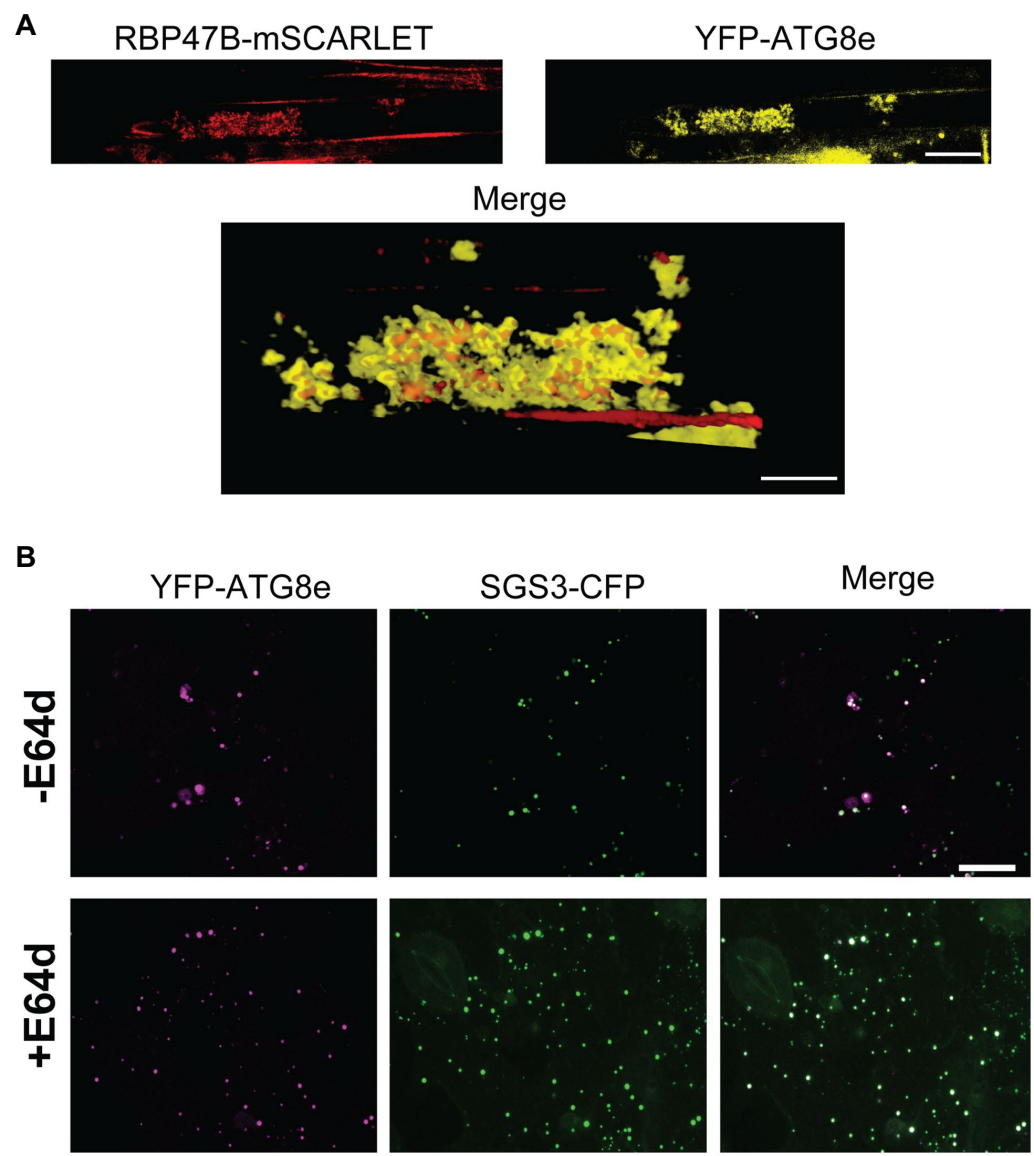
SGS3 and stress granule markers co-localize with ATG8e in E64d-induced autophagic bodies. **(A)** A transgenic *A. thaliana* reporter line with *RBP47B-mSCARLET* and *YFP-ATG8e* transgenes was subjected to 24h hypoxia in the presence of E64d. The top panel shows a representative confocal micrograph illustrating the accumulation of both signals within fluorescent clusters similar to autophagic bodies. The bottom panel represents a 3D reconstructed merged image illustrating RBP47B and ATG8e co-localization within these clusters. **(B)** Co-localization experiment with *N. benthamiana* leaves co-transfected with constructs that express SGS3-CFP and YFP-ATG8e and subjected to 24h hypoxia. The images represent 2D projections of 5–6 optical sections (2μm/section). +E64d, leaf sections were incubated with 10μM E64d. −E64d, control. Scale bars are 25μm.

Autophagic turnover can be assayed by using the cysteine protease inhibitor E64d, which causes an arrest of autophagy that results in the accumulation of cargo in clusters of autophagic bodies in the vacuole of *A. thaliana* root cells (Merkulova et al., 2014). In the presence of the autophagy inhibitor E64d, Col-0 *SGS3-CFP* reporter plants subjected to hypoxia show three-fold higher levels of SGS3-CFP foci compared to plants in the absence if E64d (Figures 4C,D). Further, the SGS3-CFP foci in E64d-treated plants showed accumulation within fluorescent clusters (Figure 4C). Closer examination of DIC and fluorescent images (Supplementary Figure S1) show that these clusters appear in the vacuole, and resemble the vacuolar autophagic bodies that accumulate in *A. thaliana* roots upon E64d autophagy arrest (Merkulova et al., 2014). Examination of an *A. thaliana* reporter line that co-expresses autophagosome (YFP-ATG8e) and stress granule (RBP47B-mScarlet) markers shows that these E64-induced clusters show strong co-localization of both signals, supporting this hypothesis (Figure 5A).

Further support for autophagy-mediated degradation of SGS3-CFP bodies during hypoxia comes from the analysis of two additional autophagy inhibitors, concamycin A and wortmannin that block distinct parts of the macroautophagy process. Similar to E64d, both inhibitors induce a 3-fold higher accumulation of SGS3-CFP bodies during extended hypoxia (Figure 4D). In contrast to the observations with Col-0, SGS3-CFP body numbers in E64d, concamycin A, and wortmannin-treated *cml38* SGS-CFP plants were indistinguishable from untreated controls (Figure 4D). Additionally, there was no apparent accumulation of clustered autophagic body-like structures in the root cells of E64d-treated *cml38* SGS3-CFP plants (Figure 4C). Overall, the data support the hypothesis that extended hypoxia triggers autophagic turnover of SGS3 bodies, and that CML38 is required for this process.

**Figure 6:**
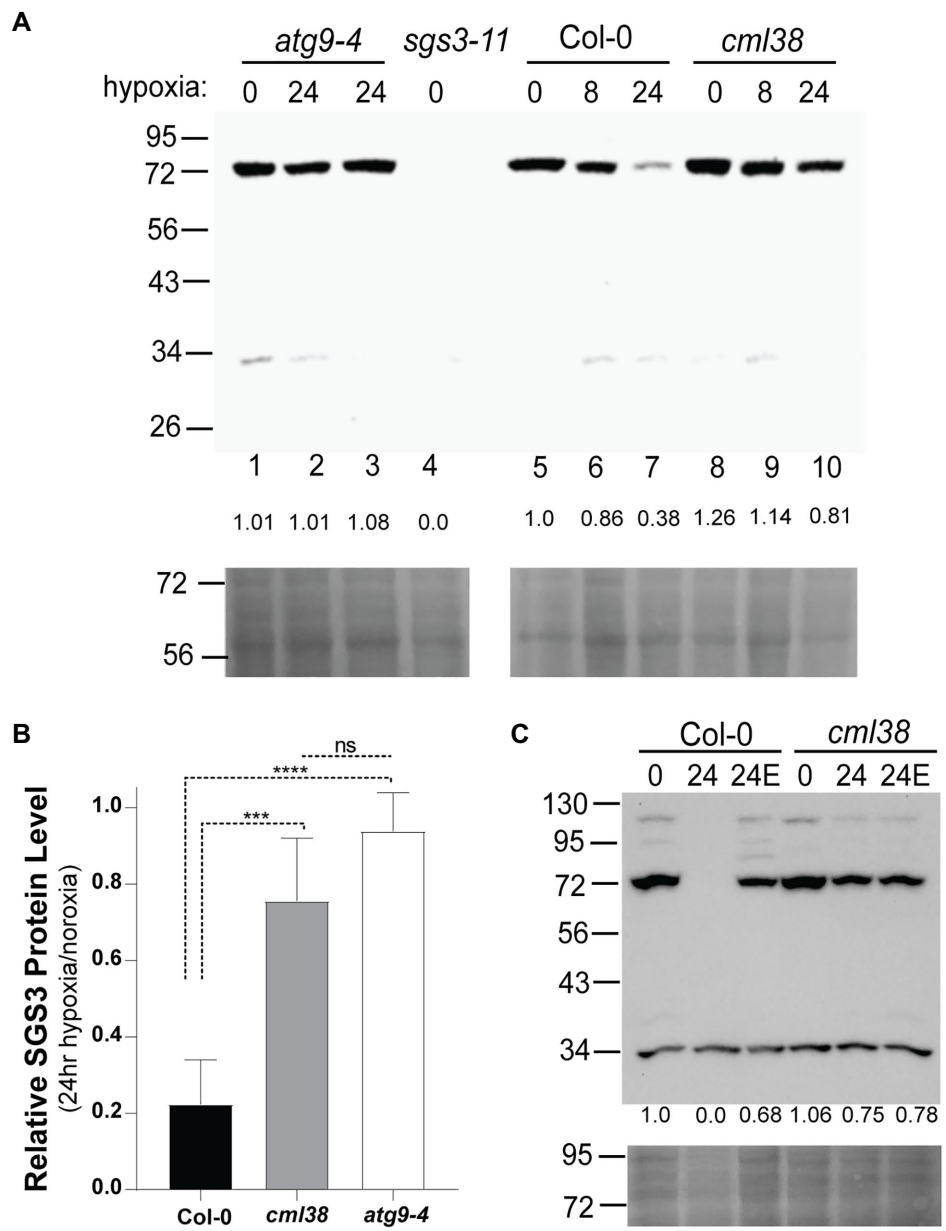
SGS3 protein levels decline during extended hypoxia *via* CML38-dependent autophagy. **(A)** SGS3 western blot (antibodies against native *A. thaliana* SGS3, AgriSera) of root extracts (top) and corresponding Ponceau-S stain (bottom) of the Col-0, *cml38*, *sgs3-11*, and *atg9-4* lines from 40-day old hydroponic *A. thaliana* plants. Each lane was loaded with 40μg total protein based on Bradford analysis. The predicted molecular weight of SGS3 is 71.97kDa. The numbers at the top of the blot indicate the duration of hypoxia treatment (8h or 24h hypoxia or 0h). Two *atg9-4* replicates of 24h samples are shown in lanes 2 and 3. The numbers below each lane show the densitometric ratio of the signal to wild type normoxia. **(B)** Relative SGS3 protein levels in 24 hypoxia-treated Col-0 (*n*=8), *cml38* (*n*=6), and *atg9-4* (*n*=3). Quantitation of the Western signal by densitometry was done followed by standardization of the 24h signal to normoxic samples. Error bars show SD. Statistical significance was assessed by one way ANOVA analysis (****, *p*<0.0001; ***, *p*<0.001; ns, not significant). **(C)** SGS3 western blot (top) and corresponding Ponceau-S stain (bottom) of root extracts of the Col-0 and *cml38* under normoxic conditions (0), 24h hypoxia (24), or 24 hypoxia treated with 10μM E64d (24E). The numbers under each lane are the densitometric ratio to wild type normoxia.

To test more rigorously whether SGS3 is subject to hypoxia-induced autophagy, we investigated the protein levels by SGS3 Western blot in Col-0, *cml38*, and the autophagy deficient line *atg9-4*. To verify the detection of endogenous SGS3 by anti-SGS3 antisera, Western blot analysis of root extracts from Col-0 and the *sgs3-11* splice site mutant line were compared (Figure 6A). A band near 72kDa was observed in Col-0 (predicted molecular weight for SGS3 is 71.97kDa), which was absent in the *sgs3-11* splice site mutant line (Figure 6A). In response to extended (24h) hypoxia, the SGS3 protein level decreased an average of 5-fold compared to the protein level under normoxia (Figure 6B). This loss of SGS3 protein was inhibited by E64d (Figure 6C). In contrast, SGS3 protein levels in the *cml38* background remain elevated during extended hypoxia (Figures 6A,B) and showed no effect of E64d (Figure 6C; Supplementary Figure S3A). Further, the differences between Col-0 and *cml38* appear to be mediated at the protein level since there is no difference in the *SGS3* transcript levels between the two genotypes (Supplementary Figure S2). Overall, the data support the results of SGS3-CFP foci experiments (Figure 4) and suggest that CML38 is necessary for the loss of SGS3 during long term hypoxia.

To determine whether the decline in the SGS3 protein level is the result of autophagy, the SGS3 protein level was assayed in the autophagy deficient line *atg9-4* (Floyd et al., 2015). In *atg9-4* roots, extended hypoxia did not affect the SGS3 protein levels (Figures 6A,B). ATG9 plays a critical role in lipid trafficking during autophagosome formation. To verify further that ATG8-mediated autophagy of SGS3 occurs during hypoxia, the SGS3 protein levels were assayed in another autophagy deficient line, *atg5* and the co-localization of SGS3 and ATG8e in *N. benthamiana* was assessed. ATG5 plays a critical role in the lipidation of ATG8 that is necessary for ATG8-mediated selected autophagy (Thompson et al., 2005). Analysis of the *atg5-1* line in response to hypoxia stress shows an unusual pattern, with the accumulation of SGS3 as a high molecular weight triplet of bands between 95 and 130kDa in addition to the 72kDa band (Supplementary Figure S3). This pattern of SGS3 bands is retained through 24h of hypoxia, whereas the SGS3 protein signal is lost in Col-0 controls (Supplementary Figure S4), supporting the requirement of autophagy for the degradation of SGS3.

To further determine whether SGS3 bodies accumulate in ATG8e-marked autophagosomes, the two proteins fused to distinct fluorescent markers were co-transfected into *N. benthamiana* and co-localization was analysed in the presence and absence of E64d (Figure 5). In contrast to *A. thaliana*, E64d treatment of tobacco cells causes the accumulation of autolysosome-like structures in the cytosol (Takatsuka et al., 2011; Merkulova et al., 2014). Co-localization analysis of co-transfected leaf disks subjected to 24 hypoxia treatment showed YFP-ATG8e marked autophagosomes and SGS3-CFP foci that showed some overlap of signal, as well as some accumulation of each marker in distinct foci (Figure 5B). This is quantitatively demonstrated from the calculation of Mander’s co-localization co-efficient (MC) for the SGS3 signal overlap with the ATG8e signal (MC=0.31, SD=0.30, *n*=32 cells). In the presence of E64d, the number of SGS3 foci increase, a stronger co-localization of SGS3 foci with ATG8e is observed (MC=0.72, SD=0.22, *n*=32 cells). This suggests the inhibition of autophagy by E64d results in accumulation of SGS3 bodies within autophagosomes/autolysosome structures that contain ATG8e. Together, the Western blot and SGS3-CFP imaging data support the hypothesis that hypoxia induces the selective autophagy of SGS3 protein and SGS3 bodies, and that CML38 is required for this process.

### CML38-Dependent Autophagy of SGS3 Bodies Requires CDC48

**Figure 7:**
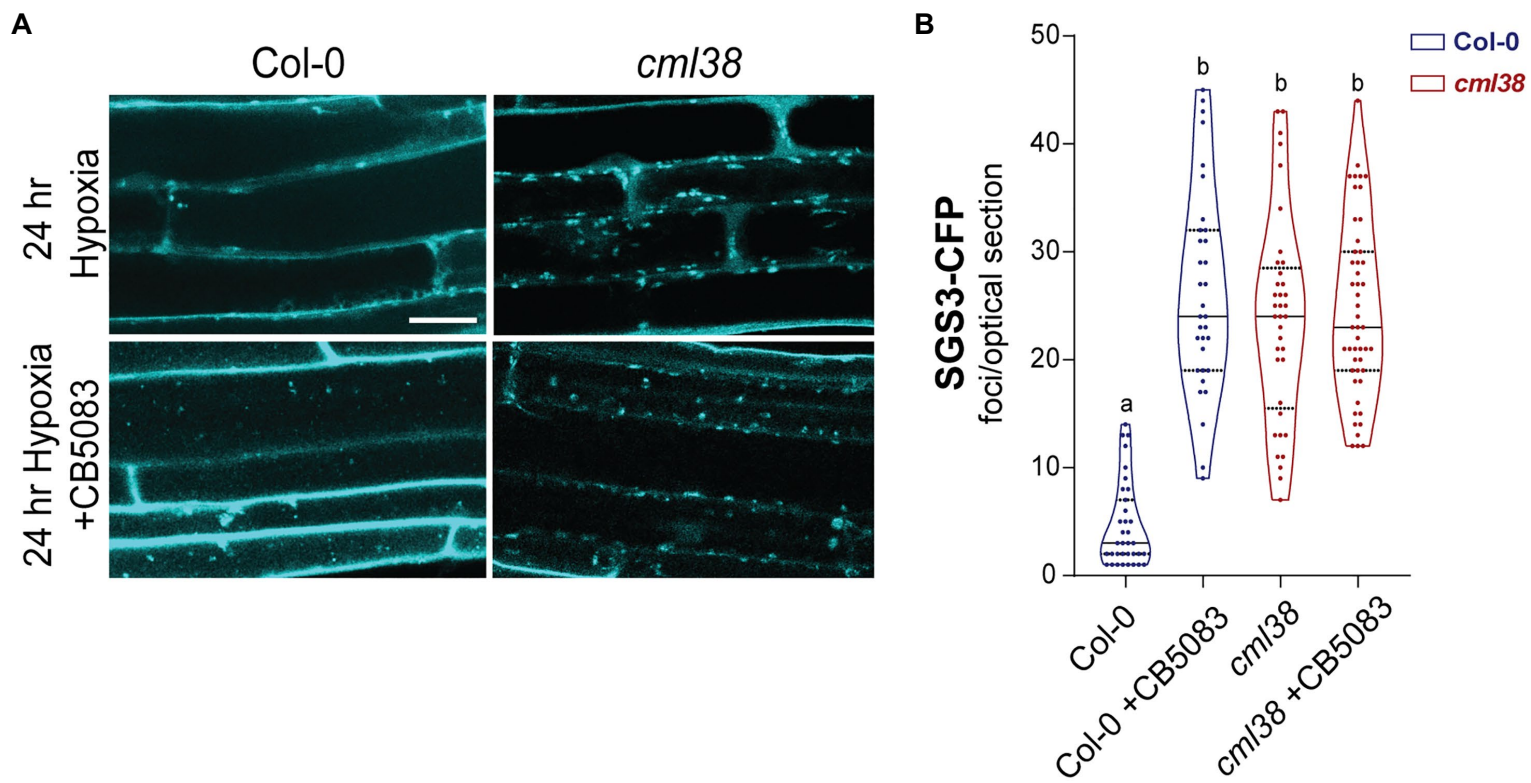
CDC48 is required for the disappearance of SGS3-CFP bodies during extended hypoxia. **(A)** Representative epidermal cell images of 10-day old *A. thaliana SGS3-CFP* Col-0 and *cml38* seedlings challenged with argon induced hypoxia for 24h in the presence or absence of 2μM CB5083. All micrographs are the same scale, and the scale bar is 25μm. **(B)** Violin plot of SGS3-CFP foci quantitation of epidermal cells treated as described in (**A**). Each data point represents a 2μm optical section from a single cell. Statistical significance was assessed by one-way ANOVA.

Previous work in yeast shows that stress granule autophagy requires CELL DIVISION CYCLE 48 (CDC48; Buchan et al., 2013). CDC48 is an AAA^+^-ATPases of the CDC48/VCP-p97 ubiquitin segregase family that recognizes and removes ubiquitinylated proteins from protein complexes (Buchan et al., 2013; Frankel et al., 2017; Ye et al., 2017). During severe stress in yeast and mammalian cells, CDC48/VCP-p97 remodels stress granules in a manner that targets them for selective autophagy (Buchan et al., 2013). The inhibitor CB5083 (Marshall et al., 2019) specifically binds the CDC48 ATPase domain and prevents ATPase activity (Tang et al., 2019). To test whether *A. thaliana* CDC48 is necessary for degradation of SGS3 bodies during sustained hypoxia, the effect of CB5083 on SGS3-CFP body accumulation was evaluated in the Col-0 and *cml38* mutant backgrounds (Figure 7).

CB5083-treated Col-0 *SGS3-CFP* reporter plants showed 5-fold higher levels of SGS3-CFP bodies in root epidermal cells at 24h hypoxia compared to the 24h hypoxic untreated controls (Figure 7). Interestingly, the number of SGS3-CFP bodies in hypoxia-challenged CB5083 treated Col-0 root cells were statistically indistinguishable from the numbers in *cml38* root cells (Figure 7B). Further, unlike Col-0 plants, CB5083 treatment showed no effect on the levels of SGS3-CFP foci in 24h hypoxia treated *cml38* plants (Figure 7B). The data suggest that both CDC48 and CML38 are necessary for SGS3-CFP body autophagy during extended hypoxia. In addition, it appears as if a CDC48-mediated granulophagy mechanism, similar to what is observed in other eukaryotic systems for stress granules, is conserved in *A. thaliana*, and this process requires the hypoxia-induced plant protein CML38.

### CML38 Recruits CDC48A to Hypoxia-Induced Cytoplasmic Granules

As noted in Figure 1, CDC48A is also a direct binding target for CML38, and previous data shows that CDC48A is a prominent component of the CML38 “interactome” identified from pulldown assays of hypoxic *A. thaliana* roots (Lokdarshi et al., 2016). Given these observations, and the fact that both proteins are required for SGS3 body autophagy, we used transient transfection experiments in *N. benthamiana* to investigate if CDC48A co-localizes with CML38 to cytoplasmic foci during hypoxia stress.

**Figure 8:**
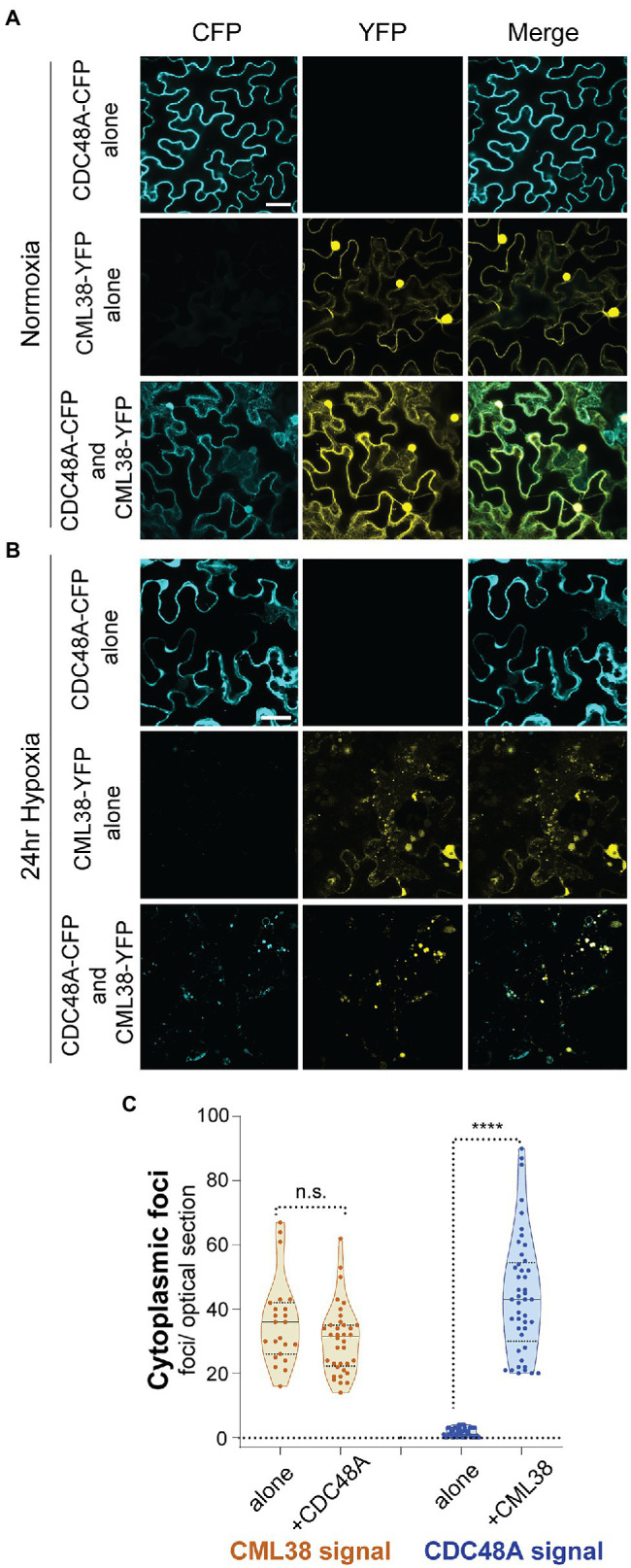
CML38 localizes CDC48A to hypoxia-induced foci in *N. benthamiana*. Representative confocal micrographs of *N. benthamiana* leaves transfected with CML38-YFP, CDC48A-CFP, or both proteins. Leaf sections were visualized 48h after infiltration. **(A)** Normoxic leaf section. **(B)** Leaf section after 24h of argon induced hypoxia. Images for (**A**,**B**) are 2D projections of 3–5 optical sections (1μm/section). **(C)** Cytoplasmic foci quantitation of 24h hypoxia-treated *N. benthamiana* leaf sections. Each data point is the number of foci in a 40μmx40μm optical section. Statistical significance was assessed by unpaired *t*-test. n.s., not significant. **** is *p*<0.0001. Scale bar is 25μm in (**A**,**B**).

Localization experiments were carried out by transfection with CDC48A-CFP alone, CML38-YFP alone, or co-expression of CDC48A-CFP and CML38-YFP (Figure 8). Under normoxia (Figure 8A) and 24h hypoxia (Figure 8B), leaf cells expressing CDC48A-CFP alone showed largely diffuse signal with no apparent localization to cytoplasmic foci (Figures 8A,B). Consistent with previous observations (Lokdarshi et al., 2016), under normoxia, CML38-YFP accumulates as diffuse signal in both the cytosol and nucleus (Figure 8A), but redistributes strongly to cytosolic stress granule foci in response to hypoxia-treatment (Figure 8B, also Figure 2). When CML38-YFP and CDC48A-CFP were co-expressed, there was little apparent change in the normoxia patterns compared to that observed when either protein is expressed alone (Figure 8A). However, the CDC48A-CFP signal showed a drastic difference under hypoxia conditions when co-expressed with CML38-YFP. Co-expression with CML38 resulted in the strong co-localization of the CDC48A-CFP signal with CML38 cytosolic foci (Figures 8B,C; Supplementary Figure S4) with the complete loss of the diffuse signal observed in CDC48A-CFP signal and nearly complete localization to the CML38 granules (Mander’s co-localization co-efficient of CDC48A-CFP to CML38-YFP signal is 0.72, Supplementary Figure S4). The data strongly suggest that CML38 co-expression results in the recruitment of CDC48A to CML38 stress granules. Based on BACTH data that shows that the two proteins interact, the observation that CML38 and CDC48A are both essential for granule autophagy, and previous evidence for CDC48 as a mediator of granulophagy in yeast and mammalian systems, we propose that CDC48A and CML38 coordinate SGS3 body granulophagy during hypoxia stress.

## Discussion

Calmodulin (CaM) and calmodulin-like (CML) proteins represent a diverse collection of calcium sensor proteins that mediate the effects of calcium signals through the binding and modulation of downstream target proteins (Chin and Means, 2000; Villalobo et al., 2019). The biological functions of CaM and CMLs are largely defined by their cadre of direct interaction targets rather than intrinsic biochemical activities associated with each protein. While previous data shows that the hypoxia calcium-sensor protein CML38 associates with stress granules, and that a number of RNA metabolism and RNA granule proteins have been identified in CML38-pull down experiments (Lokdarshi et al., 2016), the direct binding regulatory targets for CML38 have remained elusive. By using pairwise analysis of CML38 interaction with various putative protein targets in a bacterial two hybrid analysis, we were able to distinguish three proteins (SGS3, CDC48A, and DUF581-5) within the CML38 “interactome” that directly bind to CML38. CML38 interaction with two of these, SGS3 and CDC48A, appears to be involved in RNA granule homeostasis through autophagy. Potential regulatory functions of the third protein, DUF581-5, remain less clear but could participate in the energy sensing SnRK1 pathway.

### Stress Granule-Like SGS3 Bodies Are Induced by Hypoxia and Are Subject to Autophagic Turnover

SUPPRESSOR OF GENE SILENCING 3 encodes an RNA-binding protein that, together with RNA-dependent RNA polymerase 6 (RDR6), is necessary for the production of dsRNA precursors for siRNA production for secondary transgene, transposable element, and viral post transcriptional gene silencing (Peragine et al., 2004; Yoshikawa et al., 2005; Kim et al., 2021). SGS3 localizes to cytoplasmic foci termed siRNA bodies that contain the machinery of the RDR6 pathway (Kumakura et al., 2009; Jouannet et al., 2012). SGS3 has prion-like domains that trigger the formation of liquid–liquid phase separated condensates *in vitro*, and are essential for siRNA body formation and RDR6 localization to these structures *in vivo* (Kim et al., 2021). Consistent with this physical behavior, SGS3 also becomes strongly associated with cytosolic stress granules in response to heat stress (Jouannet et al., 2012). In the present work, we observe similar behavior with SGS3 showing strong localization with the stress granule marker RBP47B and CML38 during submergence stress in *N. benthamiana* transfected cells. The strong colocalization of SGS3 with stress granules indicates these two populations of cytoplasmic granules are linked during abiotic stress. It has been hypothesized that SGS3 or siRNA bodies could be involved as sites of stress granule seeding or nucleation during stress (Jouannet et al., 2012).

By using an *A. thaliana* transgenic *SGS3-CFP* reporter line, we observe that siRNA bodies are present at a low basal level in normoxic roots, but increase and accumulate in stress granule-like foci during hypoxia stress. This observation is consistent with previous finding that stress granules increase as part of the response of *A. thaliana* to hypoxia (Sorenson and Bailey-Serres, 2014; Lokdarshi et al., 2016). The increase in stress granules during hypoxia is part of the global suppression of mRNA translation to conserve resources and redirect gene expression to favor the core hypoxia proteins (Branco-Price et al., 2008; Mustroph et al., 2009; Lee and Bailey-Serres, 2021).

However, as the period of hypoxia stress is extended, SGS3 foci and the cellular levels of SGS3 protein based on Western blot analysis both decline. In contrast, Q-PCR analysis shows that transcript levels of *SGS3* and *RDR6* actually increase in response to this stress (Supplementary Figure S2), suggesting that the reduction of SGS3 bodies and protein levels are regulated at a posttranscriptional level. Further, the data show that there is a distinction between the SGS3/stress granule body response to short term and long term hypoxia. The time dependence of the decrease in hypoxia-dependent SGS3 bodies coincides with previous observations that extended submergence of *A. thaliana* induces the autophagy pathway, which is essential for an optimal response to sustained anaerobic stress (Chen et al., 2015). In the present study, evidence is provided that long-term hypoxia stress induces SGS3 protein degradation through the autophagy of SGS3/stress granule bodies. The induction of SGS3 autophagy during long term hypoxia, but not during early phases of this stress, may be due to the transition from carbohydrate utilization and fermentative metabolism to more acute carbohydrate starvation and the need to recycle cellular resources (Cho et al., 2021) including stress granule aggregates. Additionally, the regulation of RNA populations and gene expression change with the severity and duration of the stress (Lee and Bailey-Serres, 2021), and the degradation of SGS3/stress granule bodies may also be part of this regulation.

### Autophagy of SGS3 Bodies Requires CML38 and CDC48A

CML38 and SGS3 are interacting proteins that show strong co-localization within hypoxia-induced granules. The potential biological significance of this interaction comes from the observation that *cml38* mutants no longer show SGS3 protein and SGS3 body turnover during extended hypoxia, suggesting that CML38 is essential for SGS3 body autophagy. In this regard, CML38 is similar to its homolog from *Nicotiana*, rgsCaM, which binds directly to SGS3, and triggers the autophagy of the protein and the reduction in siRNA bodies in *N. benthamiana* (Li et al., 2017). RgsCaM-induced autophagy prevents the RDR6-dependent production of dsRNA templates for secondary siRNA molecules, and accounts for rgsCaM’s suppression of silencing activity (Li et al., 2017). The present findings that CML38 is a direct binding target for SGS3, and is necessary for SGS3 autophagy in hypoxic *A. thaliana*, suggests that CML38 may share this rgsCaM function. In support of this hypothesis, CML38 is indistinguishable from rgsCaM and the potyviral suppressor protein HC-Pro in its ability to suppress silencing in the *N. benthamiana* 16C line (Supplementary Figure S5), further suggesting a conserved function for the rgsCaM family in PTGS and RNA granule regulation.

While CML38 has rgsCaM-like silencing suppression activity in the 16C line, it remains unresolved whether it has a role in secondary siRNA regulation during severe hypoxia. Analysis of the small RNA population in hypoxia-stressed *A. thaliana* show that secondary siRNAs such as trans-acting siRNAs (tasiRNAs) are a minor component compared to miRNAs (Moldovan et al., 2010). Analysis of tasiRNAs in 24h hypoxic Col-0 and *cml38* roots by Q-PCR analysis show little differences with tasiRNAs present at the limits of detection in both lines (data not shown). Rather than regulation of RDR6-dependent siRNA pathways, SGS3 may instead play a role as a hypoxia stress granule-associated protein, and that CML38 interaction may target SGS3/stress granule bodies for autophagy under extreme low oxygen stress, either to recycle cell components, regulate RNA populations in granules, or clear damaged cellular components.

The autophagic degradation of RNA granules in yeast and mammalian systems (referred to as “granulophagy”) requires the action of the AAA^+^-ATPase CDC48/p97 (Buchan et al., 2013; Protter and Parker, 2016; Alberti et al., 2017; Frankel et al., 2017). CDC48/p97 is an abundant and ubitquitous chaperon protein with ubiquitin segregase activity (Ye et al., 2017). CDC48/p97 recognizes and binds ubiquitinylated proteins, generally through the action of accessory co-factor proteins. It mediates the ATP-dependent removal of ubiquitinylated proteins from supramolecular complexes, and promotes their degradation by autophagy or the Ub-proteosome pathway (Ye et al., 2017). In the case of stress granule granulophagy, ubiquitin modification of stress granules followed by CDC48/p97 remodeling is proposed to be an essential step that leads to granule autophagy and granulostasis (Protter and Parker, 2016; Alberti et al., 2017). The finding that CDC48/p97 inhibitor CB5083 inhibits the hypoxia-dependent decline in SGS3 bodies suggests that this mechanism is conserved in plants.

Consistent with this proposed connection between CDC48 and SGS3 body turnover, SGS3 is a target for ubiquitination which leads to degradation by either the Ub-proteosome pathway (Liu et al., 2019) or autophagy (Li et al., 2017). In the case of heat stress, SGS3 ubiquitination and subsequent degradation is triggered by an E3 ligase encoded by *SGS3 INTERACTING PROTEIN I* (*SGIP1*; Liu et al., 2019). SGIP1 catalyzes the ubiquitination of SGS3 resulting in the accumulation of higher molecular weight forms of the protein (Liu et al., 2019). Interestingly, in this study we observe a similar triplet of higher molecular weight SGS3 forms in the *atg5-1* autophagy deficient mutant, further supporting a potential link between autophagy and SGS3 ubiquitination.

### CML38 Interacts With CDC48A and Leads to Its Localization to Hypoxia-Induced Granules

Unlike Col-0 plants, CB5083 shows no effect on SGS3 bodies in the *cml38* background, suggesting that CDC48 and CML38 work in a coordinate fashion in granule autophagy. The mechanism through which CDC48 and CML38 could regulate SGS3 and stress granule autophagy is not yet clear, but observations from previous studies and the present work provide potential leads. The predominant isoform of CDC48 in *A. thaliana*, CDC48A, is a major component identified from CML38 pulldown experiments (Lokdarshi et al., 2016), and here we show that CDC48A is a direct binding target for CML38 by BACTH analysis (Figure 1). Moreover, transient transfection experiments show that CDC48A localization does not change significantly during hypoxia. However, upon co-transfection with CML38 it strongly (>70% of the signal based on Mander’s co-localization analysis) localizes to hypoxia-induced CML38 granules (Figure 8). Based on these collective observations, we hypothesize that CML38 binds and localizes CDC48A to hypoxia-stress induced stress granules. Further, the fact that CDC48 inhibitors have no effect on granule accumulation in *cml38* plants (Figure 7) suggests that the CDC48A:CML38 interaction may also be necessary for CDC48-induced granulophagy. In this regard, CML38 may function similar to other CDC48/p97 adaptor/co-factor proteins that recruit this AAA^+^-ATP segregase to specific subcellular structures and substrates, often as part of a multi-protein complex (Ye et al., 2017).

### Additional CML38 Regulatory Functions During Low Oxygen Stress

Based on CML38 pulldown and BACTH interaction, the SnRK1 binding protein DUF581-5 could represent an additional regulatory target for CML38. DUF581-5 (also known as FTZ13) is a member of a plant-specific family of FCS C2-C2 zinc finger proteins that bind to the catalytic subunits of the SnRK1 protein kinase (Nietzsche et al., 2014). SnRK1 signaling coordinates responses to changes in cellular energy status, and works by repressing energy consuming pathways and redirecting resources during stress (Baena-González et al., 2008; Crepin and Rolland, 2019). Related to hypoxia-stress signaling, activation of SnRK1 modulates selective core hypoxia response protein translation as well as other responses to carbohydrate starvation during long-term hypoxia (Cho et al., 2019, 2021). SnRK1 also works coordinately with Target of Rapamycin (TOR) kinase pathways to regulate autophagy in response to abiotic stress (Signorelli et al., 2019). DUF581 proteins are induced in response to numerous abiotic stresses, including hypoxia (Nietzsche et al., 2014), and have been proposed to serve as negative feedback regulators of SnRK1 signaling by binding and reducing the levels of SnRK1 protein by an unknown mechanism (Jamsheer et al., 2018; Crepin and Rolland, 2019). The finding that CML38 binds to DUF581-5 suggests another potential level of regulation of this hypoxia and autophagy-associated protein kinase that merits further investigation.

### Conclusion and Working Model

**Figure 9:**
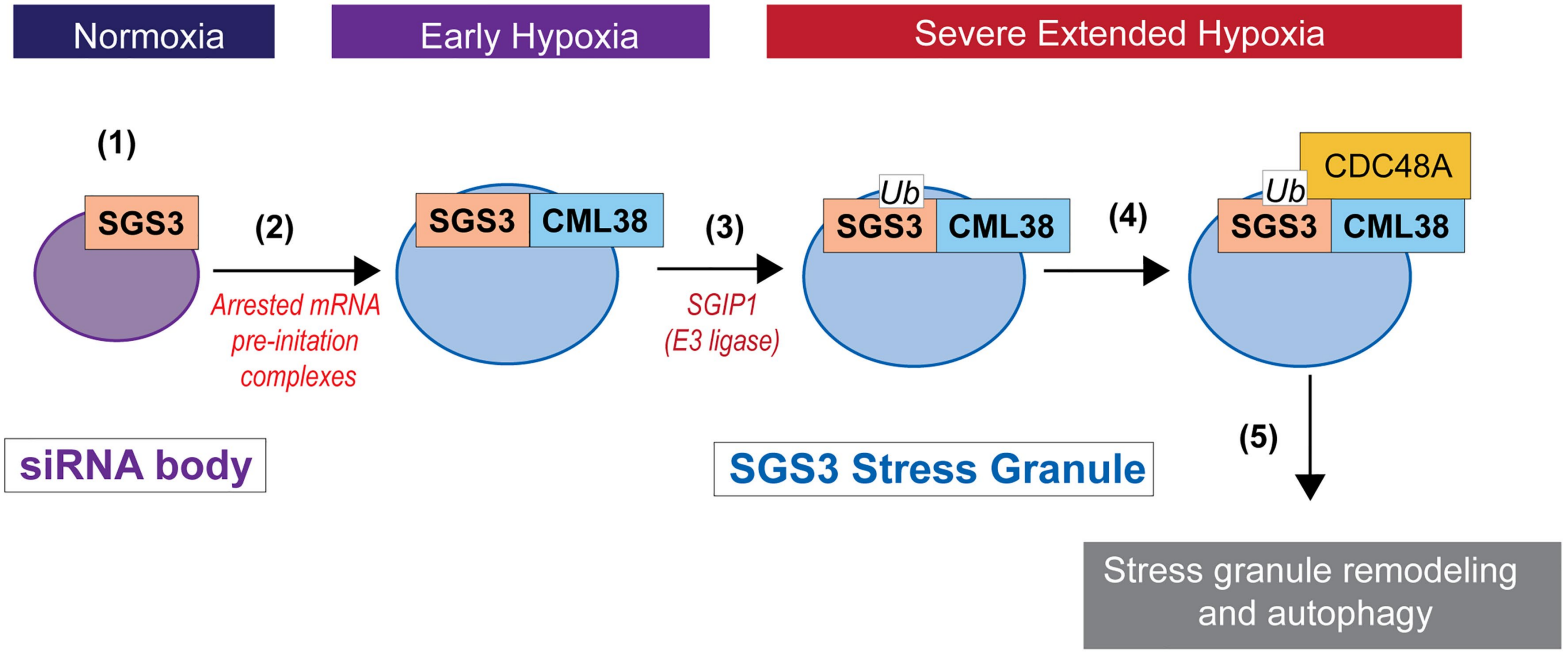
Working model for SGS3 granule regulation by CML38. (1) SGS3 phase separates and localizes to cytosolic siRNA bodies under normoxic conditions. (2) Hypoxia stress results in a low energy state that triggers translational arrest and the concomitant accumulation of mRNA into stress granules that proliferate under this stress. SGS3 accumulates within stress granules. The core-hypoxia response protein CML38 is induced and localizes to SGS3-associated stress granules where it directly interacts with SGS3. (3) The cell experiences carbon starvation during severe extended hypoxia stress that leads to the induction of autophagy. SGS3 is tagged for autophagy degradation by conjugation to ubiquitin catalyzed by an E3 ubiquitin ligase similar to SGS3 INTERACTING PROTEIN 1 (SGIP1; Liu et al., 2019). (4) CML38 recruits CDC48A to stress granules. CDC48A remodels ubiquitinylated proteins within the stress granule, including ubiquitin-tagged SGS3. (5) Stress granule protein complexes remodeled by CDC48A are targeted for autophagic turnover.

*CALMODULIN-LIKE 38* is among the core-hypoxia genes that are selectively transcribed and translated during low oxygen stress, is the only EF hand protein that is induced by hypoxia stress, and is essential for optimal response to low oxygen stress in *A. thaliana* (Lokdarshi et al., 2016). In the present study, we show SGS3 protein is a direct binding target for CML38, that SGS3 stress granule-like bodies are degraded by autophagy by a CML38-dependent mechanism during extended low oxygen stress, and that this process appears to involve the CDC48A AAA^+^-ATPase. This provides a functional link between the unique hypoxia-induced calcium sensor CML38 and stress granule homeostasis that may be another facet of ribostasis that conserves resources during the severe energy crisis associated with extended hypoxia. A working model for how these components coordinate RNA granule autophagy during extended hypoxia is presented in Figure 9. Key elements of this model remain to be tested including: (1) the biochemical and structural nature of CML38 interaction with these targets; (2) the function of calcium as a sensor during the process and the nature of the calcium signal; and (3) the molecular mechanism that connects CML38 to selective granule autophagy.

## Data Availability Statement

The raw data supporting the conclusions of this article will be made available by the authors, without undue reservation.

## Author Contributions

SF designed and performed the majority of the experiments and coordinated the contributions of other authors. SF and DR wrote the manuscript. WC designed and performed *Nicotiana* RBP47B-CML38, SGS3-CML38, and SGS3-RBP47B colocalization experiments. DR supervised and conceived the project, provided support and supervision of personnel, was involved in the design of experiments, and the analysis and interpretation of data. All authors contributed to the article and approved the submitted version.

## Funding

This work was supported in part by National Science Foundation grant MCB-1121465 and support from the Charles Postelle Professorship fund (University of Tennessee-Knoxville) to DR.

## Conflict of Interest

The authors declare that the research was conducted in the absence of any commercial or financial relationships that could be construed as a potential conflict of interest.

## Publisher’s Note

All claims expressed in this article are solely those of the authors and do not necessarily represent those of their affiliated organizations, or those of the publisher, the editors and the reviewers. Any product that may be evaluated in this article, or claim that may be made by its manufacturer, is not guaranteed or endorsed by the publisher.
