# Supplementary material for: Arabidopsis CALMODULIN-LIKE 38 Regulates Hypoxia-Induced Autophagy of SUPPRESSOR OF GENE SILENCING 3 Bodies
Source: Front Plant Sci. 2021 Sep 8;12:722940. doi: 10.3389/fpls.2021.722940 (PMC8456008; doi:10.3389/fpls.2021.722940)
Supplement: Supplementary file 1 [file Data_Sheet_1.PDF]

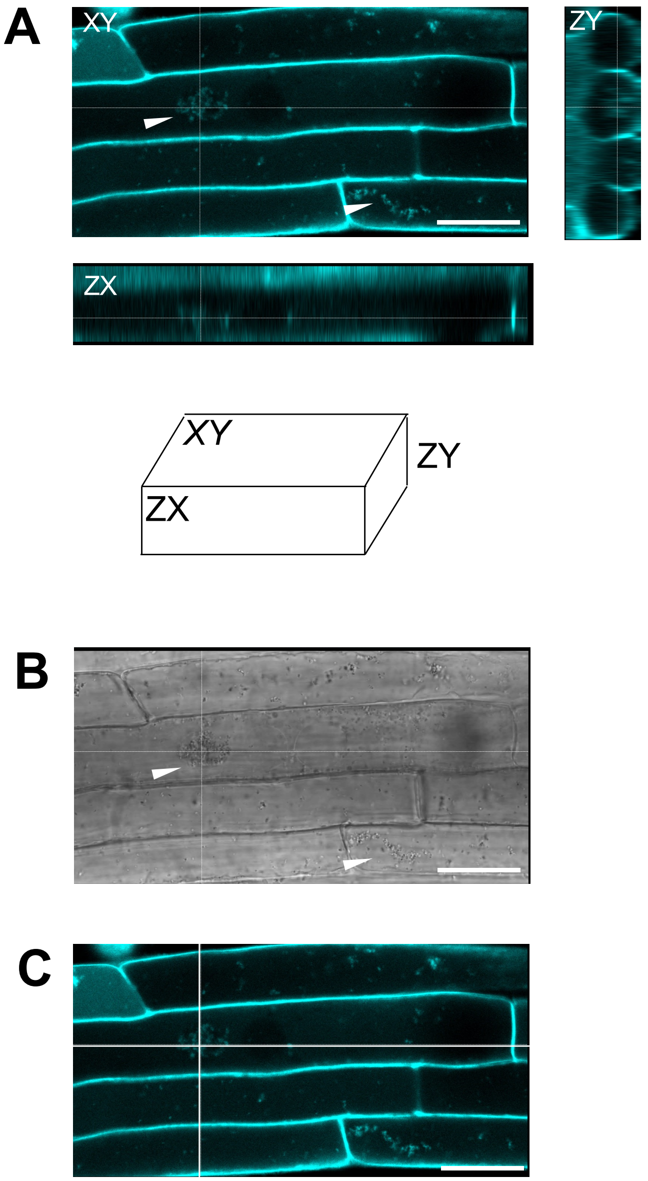

Supplemental Figure S1. SGS3-CFP foci accumulate within vacuolar autophagic body-like clusters with E64d treatment. A. (*Top)* SGS3-CFP fluorescence signal in ten day old *35s:SGS3-CFP* Col-0 seedlings at 24 hr hypoxia, treated with 10 μM E64d. Autophagic body-like clusters were visualized by orthogonal sectioning of the X Y plane, composite z-stack images were assembled to visualize the ZX and ZY planes to show location of the cluster within the cell. Images in the X, Y, and Z planes were assembled and orthogonal sections generated in Leica LAS X software from 20 optical sections (1 μm each optical section). (*Bottom)* Diagram of XY, ZX, and ZY planes. **B.** DIC micrograph of A. Arrow head indicates vacuolar autophagic body-like clusters. **C.** Cross sections indicated orthogonal sections are denoted by the white line. Scale bar is 25 μm.

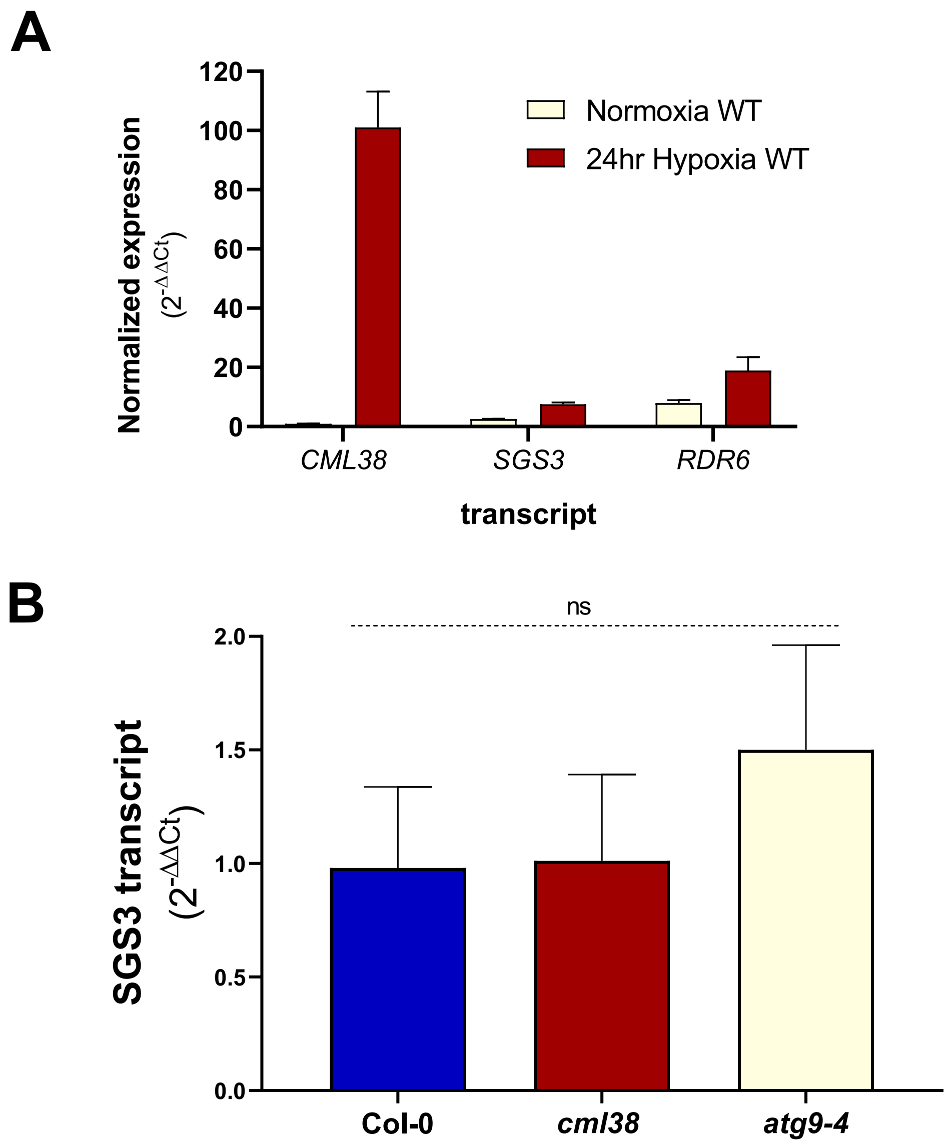

Supplemental Figure S2. ***SGS3*** **transcripts in** ***A. thaliana*** **roots at 24 hr hypoxia. A.** Q-PCR analysis of *CML38, SGS3, and RDR6* transcript levels in roots of 40-day old *Col-0 A. thaliana* plants, before hypoxia challenge (normoxia) and after 24 hr hypoxia induced by argon gas. **B.** Comparison of *SGS3* transcript levels in the roots of wild type (Col-0), *cml38* and *atg9-4* seedlings subjected to 24 hr hypoxia. UBQ10 was used as the reference transcript for each sample. In Panel A, the relative expression is normalized to normoxic levels of *CML38* (error bars represent SD of n=4 determinations). In panel B, the *SGS3* transcript levels were normalized to the average expression in 24 hr hypoxia treated Col-0 roots. Biological replicates: Col-0, n=6; *cml38,*n = 5; *atg9-4* n=5.

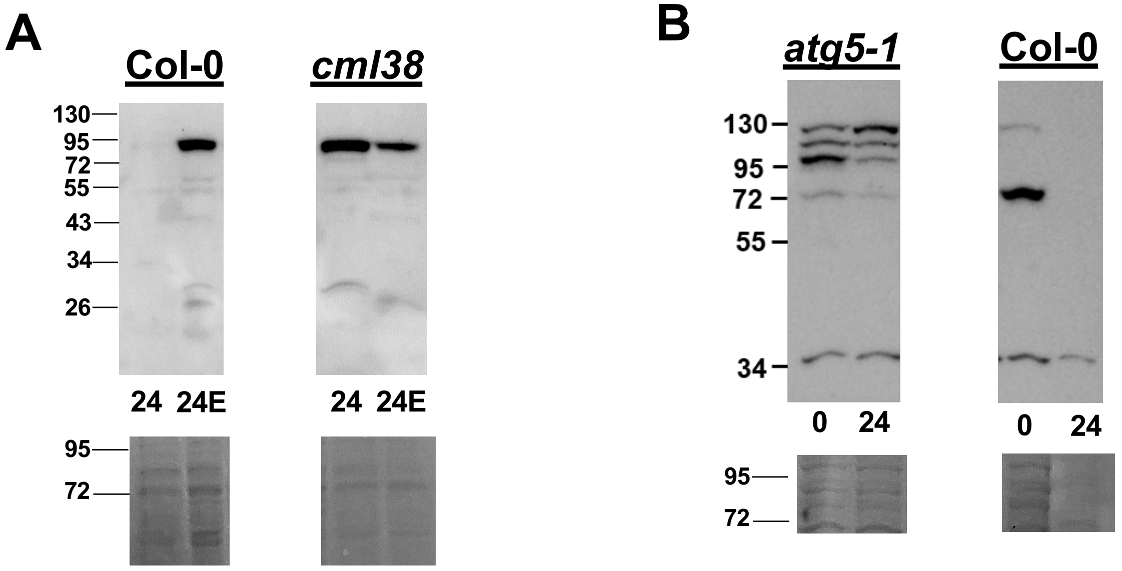

Supplemental Figure S3. SGS3 protein degradation during extended hypoxia is inhibited in *atg5* plants and in Col-0 plants treated with E64d. (A) (*Top*) SGS3-CFP Western blot (antibodies against GFP, AgriSera) and corresponding loading control image (Ponceau-S stain, *bottom*) of root extracts of *SGS3-CFP* Col-0 and *cml38* lines from 10 day old *A. thaliana* seedlings. The numbers below the blot indicate the duration of hypoxia treatment (**24** hr hypoxia) and E64d treatment (**24E**). Each lane was loaded with 40 µg total protein. The predicted molecular weight of SGS3-CFP is 98.89 kDa. **(B)** (*Top*) SGS3 Western blot (antibodies against native *A. thaliana* SGS3, AgriSera) and corresponding loading control image (Ponceau-S stain, *bottom*) of root extracts of Col-0 and *atg5-1* lines from 10 day old *A. thaliana* seedlings. Each lane was loaded with 40 µg total protein based on Bradford analysis. The numbers below the blot indicate the duration of hypoxia treatment (**24** hr hypoxia or **0** hr).

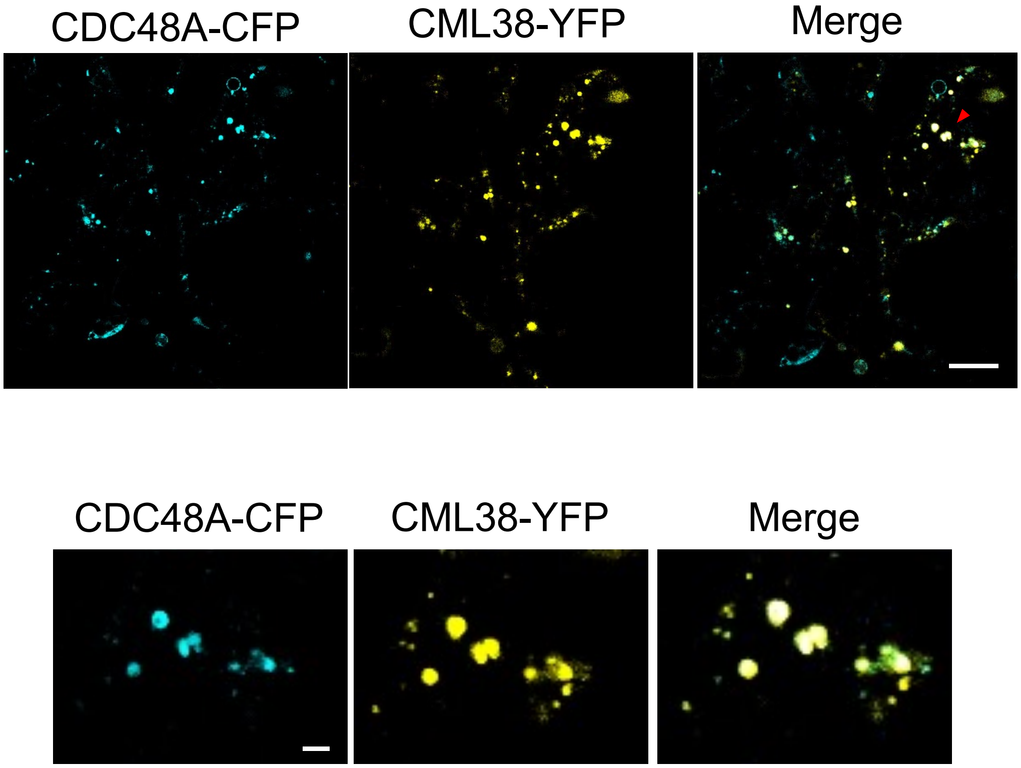

Supplemental Figure S4. Co-localization of CDC48A and CML38 in cytosolic granule-like structures. (*Top)* Confocal micrograph of CML38-YFP and CDC48A-CFP from co-expression experiments. *N. benthamiana* leaf sections were subjected to 24 hr hypoxia. The images are taken from Figure 8B bottom panel. *(Bottom)* Magnification of the granules designated by the red arrowhead in the top merge micrograph. Manders Colocalization Coefficients (MCC) at 24 hr hypoxia: $MCC_{CDC48A:CML38}$ = 0.72, SD = 0.14; $MCC_{CML38:CDC48A}$ = 0.44, SD = 0.19; n=30. Scale bar for *top* is 25 µm, *bottom* is 5 µm.

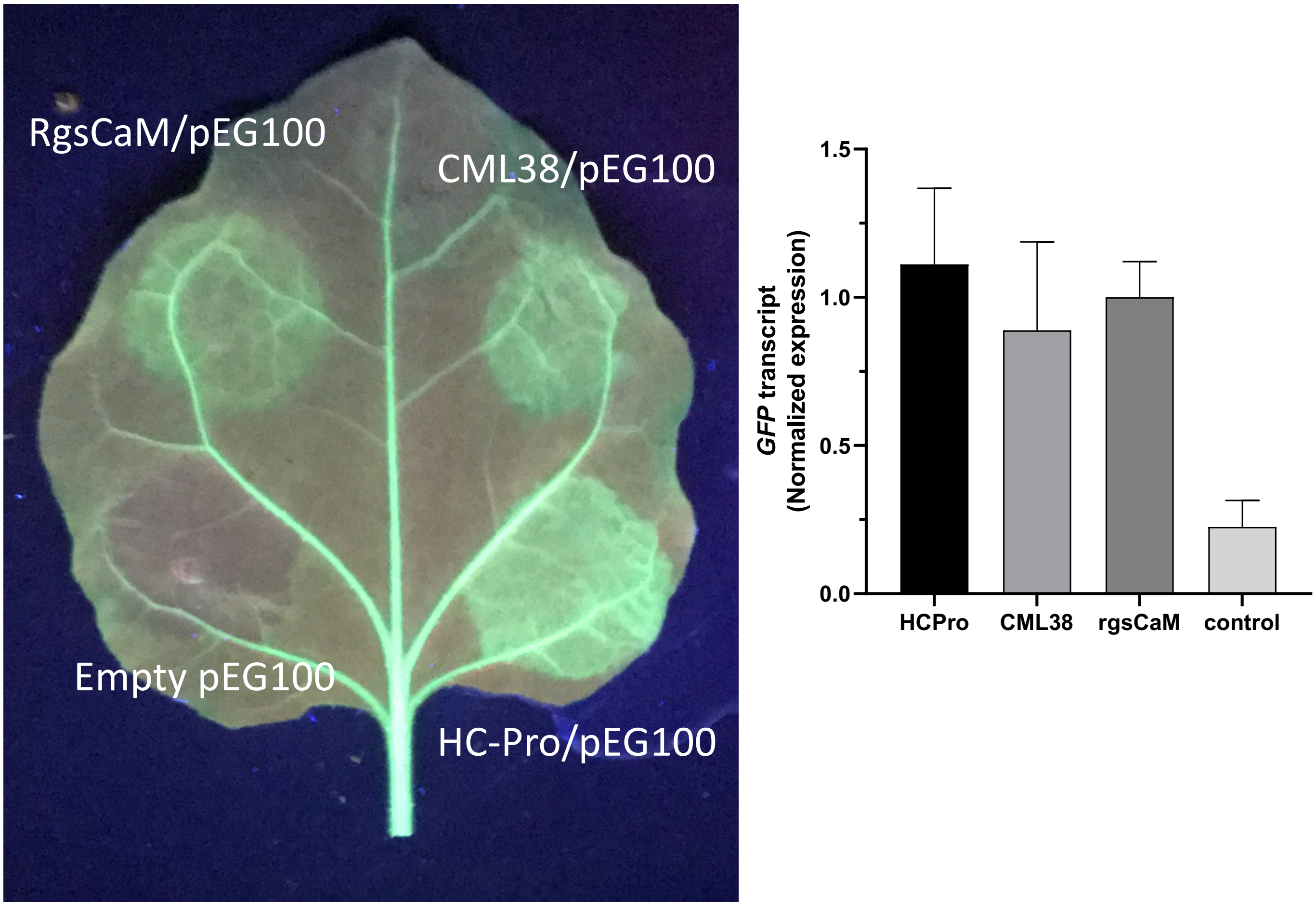

Supplemental Figure S5. *CML38* suppresses silencing in *Nicotiana benthamiana* 16C lines. *Nicotiana benthamiana* 16C leaves were transfected with *A. tumefaciens*, as described in materials and methods, containing expression constructs for turnip mosaic viral HC-Pro (positive control), *A. thaliana* CML38, *N. tabacum* RgsCaM, or empty vector negative control (see Materials and Methods). GFP fluorescence was assayed by UV light irradiation 5 days after infiltration. The histogram shows the results of Q-PCR analysis (n=4) of *GFP* mRNA. Each experiment pooled 3 leaf disks (1 cm diameter each) collected from the site of infiltration, 5 days after infiltration. *N. benthamiana* samples were normalized using *NbActin*.
