# Supplementary material for: Arabidopsis CALMODULIN-LIKE 38 Regulates Hypoxia-Induced Autophagy of SUPPRESSOR OF GENE SILENCING 3 Bodies
Source: Front Plant Sci. 2021 Sep 8;12:722940. doi: 10.3389/fpls.2021.722940 (PMC8456008; doi:10.3389/fpls.2021.722940)
Supplement: Supplementary file 2 [file Data_Sheet_2.PDF]

Table S1 Oligonucleotides used in this study.

Primer sequence (5' to 3')	Orientation	Construct (Experiment)
**CAAAAAAGCAGGCTTA**ATGAGTTCTAGGGCTGGTCC	F	*attB1- SGS3* (SGS3 reporter construct)
**CAAGAAAGCTGGGTT**ATCATCTTCATTGTGAAGGCC	R	*SGS3-attB2* (SGS3 reporter construct)
**GGGGACAAGTTTGTACAAAAAAGCAGGCTTA** ATGTCTACCCCAGCTGAATCTTCA	F	*attB1-CDC48A* (CDC48A reporter construct)
**GGGGACAACTTTGTA CAAGAAAGCTGGGTT** ATTGTAGAGATCATCATCGTCCCCAG	R	*CDC48A-attB2* (CDC48A reporter construct)
**CAAAAAAGCAGGCTTA**ATGAATAAAGGAAGCATCTTTAAGATG	F	*attB1-ATG8e* (*YFP-ATG8e* construction)
**CAACTTTGTATAGAAAAGTTGGGTGCT**ATTAGATTG AAGAAGCACCGAATG	R	*ATG8e*-Stop-*attB4* (*YFP-ATG8e* construction)
GAC<u>TCTAGA</u>ATGCAGACAACCAACG	F	*Xba*I-*RBP47B*
GAC<u>CCCGGG</u>GCTAGC ATTCTCCCCATG	R	*RBP47B-Xma*I
ATCTTCTAGAGATATCACTAGTCAACATGGTGGAGCACGA	F	*CaMV35s promoter* (for genotyping plant lines)
CGAC<u>TCTAGA</u>GATGAAGAATAATACTCAA	F	*Xba*I-*CML38* (BACTH)
CTTA<u>GGTACC</u>CGGCGCATCATAAGAGCAAA	R	*CML38-Kpn*I (BACTH)
GA<u>CTCTAGA</u>GATGTCTACCCCAGCTGAA	F	*Xba*I-*CDC48A* (BACTH)
TTA<u>GGTACC</u>CGATTGTAGAGATCATCATC	R	*CDC48A-KPN*I (BACTH)
GAC<u>TCTAGA</u>GATGAGTTCTAGGGCTGGTCCA	F	*Xba*I-*SGS3* (BACTH)
TTA<u>GGTACC</u>CGATCATCTTCATTGTGAAGGCC	R	*SGS3-Kpn*I (BACTH)
GAC<u>TCTAGA</u>GATGTCTGAAGTTGAGTACCG	F	X*ba*I-*GRP8* (BACTH)
TTA<u>GGTACC</u>CGCCAGCCGCCACCACCGCCTC	R	*GRP8-Kpn*I (BACTH)
CGAC<u>TCTAGA</u>GATGGCGTCCGGTGATGTT	F	*Xba*I-*GRP7* (BACTH)
CTTA<u>GGTACC</u>CGCCATCCTCCACCACCACCGCT	R	*GRP7-Kpn*I (BACTH)
GAC<u>TCTAGA</u>GATGGCAGGATCCGCACCG	F	*Xba*I-*eIF4A* (BACTH)
CTTA<u>GGTACC</u>CGCAGCAAATCAGCCACGTT	R	*eIF4A -Kpn*I (BACTH)
GAC<u>TCTAGA</u>GATGATACTAAGCAAGAGA	F	*Xba*I-*DUF581-5* (BACTH)
CTTA<u>GGTACC</u>CGAAATACGAATACTCCGGC	R	*DUF581-5 -Kpn*I (BACTH)
**GGGGACAAGTTTGTACAAAAAAGCAGGCTTA ATGGAATCGGTT**TCTGTACCTAG	F	*attB1-RgsCaM* (Construct for silencing assay)
**GGGGACAACTTTGTACAAGAAAGCTGGGTT** ACTTGTCATCATAACTTTGAACTCATC	R	*RgsCaM-attB2* (Construct for silencing assay)
**CAAAAAAGCAGGCTTA**ATGAAGAATAATA CTCAACCTCAATC	F	*attB1-CML38* (Silencing assay and reporter construct)

**CAAGAAAGCTGGGTT**GCGCATCATAAGAGCAAAC	R	*CML38-attB2* (Silencing assay and reporter construct)
**CAAAAAAGCAGGCTTA**ATGAGTGCAGCAGGAG	F	*attB1-HC-Pro* (Construct for silencing assay)
**CAAGAAAGCTGGGTT**TGCAGCTCCGACGC	R	*HC-Pro-attB2* (Construct for silencing assay)
TGGCAGCTTGGATAGCTTGT	F	*SGS3* (qPCR primer)
CTAGTAGAGGGTGCAGGTT	R	*SGS3* (qPCR primer)
AGAAACTCATCCCTCCCAACAG	F	*RDR6* (qPCR primer)
CCAACTGCTCATTCGCCAAG	R	*RDR6* (qPCR primer)
CACACTCCACTTGGTCTTGCGT	F	*Ubq10* (qPCR primer)
TGGTCTTTCCGGTGAGAGTCTTCA	R	*Ubq10* (qPCR primer)
CAGCCGGAGAGATACAACAACA	F	*CML38* (qPCR primer)
CTCTTTTGAAGCTCTTCTGGTGAGA	R	*CML38* (qPCR primer)
GATGAAGATACTCACAGAAAGA	F	*N. benthamiana Actin* (qPCR primer)
GTGGTTTCATGAATGCCAGCA	R	*N. benthamiana Actin* (qPCR primer)
GGAGAGGGTGAAGGTGATGCAAC	F	*GFP* (qPCR primer)
CCGTCGTCCTTGAAGAAGATGGT	R	*GFP* (qPCR primer)

*Restriction sites are denoted by the underlined bases.
**Gateway Cloning Recombination sites (Invitrogen) are denoted by bold bases.
