# Supplementary material for: Arabidopsis CALMODULIN-LIKE 38 Regulates Hypoxia-Induced Autophagy of SUPPRESSOR OF GENE SILENCING 3 Bodies
Source: Front Plant Sci. 2021 Sep 8;12:722940. doi: 10.3389/fpls.2021.722940 (PMC8456008; doi:10.3389/fpls.2021.722940)
Supplement: Supplementary file 3 [file Data_Sheet_3.PDF]

<u>Table S2</u> Constructs made in this study

Construct	Source	Identifier	Application
pEarleyGate 102 *SGS3*	AT5G23570	*SGS3-CFP*	*A.thaliana* transgenic line, *N. benthamiana* co-localization.
pEarleyGate 102 *CDC48A*	AT3G09840	*CDC48A-CFP*	*N. benthamiana* co-localization
pBIN19 *RBP47B*	AT3G19130	*RBP47B-mSCARLET*	*A.thaliana transgenic line*
pEarleyGate 104 *ATG8e*	AT2G45170	*YFP-ATG8e*	*A.thaliana* transgenic line, *N. benthamiana* co-localization
pKNT25 *CML38*	AT1G76650	*CML38*/pKNT25	BACTH
pUT18C *CDC48A*	AT3G09840	*CDC48A*/pUT18C	BACTH
pUT18C *SGS3*	AT5G23570	*SGS3*/pUT18C	BACTH
pUT18C *GRP8*	AT4G39260	*GRP8*/pUT18C	BACTH
pUT18C *GRP7*	AT2G21660	*GRP7*/pUT18C	BACTH
pUT18C *eIF4A*	AT1G54270	*eIF4A*/pUT18C	BACTH
pUT18C *DUF581-5*	AT1G74940	*DUF581-5*/pUT18c	BACTH
pEarleyGate 100 *CML38*	AT1G76650	*CML38/*pEG100	Silencing suppression assay
pEarleyGate 100 *NtrgsCaM*	AF329729.1	*NtrgsCaM/*pEG100	Silencing suppression assay
pEarleyGate 100 *HC-Pro*	EF028235	*HC-Pro/*pEG100	Silencing suppression assay
